# Trends and inequities in adolescent childbearing in Latin American and Caribbean countries across generations and over time: a population-based study

**DOI:** 10.1016/S2352-4642(23)00077-9

**Published:** 2023-06

**Authors:** Antonio Sanhueza, Janaína Calu Costa, Oscar J Mújica, Liliana Carvajal-Velez, Sonja Caffe, Cesar Victora, Aluísio J D Barros

**Affiliations:** a Pan-American Health Organization, Washington, DC, USA; b International Center for Equity in Health, Federal University of Pelotas, Pelotas, Brazil; c United Nations Children's Fund, New York, NY, USA; d Department of Global Public Health, Karolinska Institutet, Stockholm, Sweden

## Abstract

**Background:**

Latin America and the Caribbean present the second highest adolescent fertility rate in the world, only after sub-Saharan Africa, and have reached the third position globally in the incidence of motherhood in adolescence. We aimed to explore trends and inequities in adolescent childbearing in the region.

**Methods:**

We used nationally representative household surveys from Latin American and Caribbean countries to address trends in early childbearing (proportion of women having their first livebirth before age 18 years) over generations and in adolescent fertility rates (AFRs; livebirths per 1000 women aged 15–19 years) over time. For early childbearing, we analysed the most recent survey conducted since 2010 from 21 countries (2010–20); for AFR, we analysed nine countries with two or more surveys, with the most recent being conducted from 2010 onwards. For both indicators, variance-weighted least-square regression was used to estimate the average absolute changes (AACs) at the national level and by wealth (bottom 40% *vs* top 60%), urban versus rural residence, and ethnicity.

**Findings:**

Among 21 countries studied, we noted a decrease in early childbearing along generations in 13 of them, with declines varying from –0·6 percentage points (95% CI –1·1 to –0·1) in Haiti to –2·7 percentage points (–4·0 to –1·4) in Saint Lucia. We observed increases over generations in Colombia (1·2 percentage points [0·8 to 1·5]) and Mexico (1·3 percentage points [0·5 to 2·0]) and no changes in Bolivia and Honduras. The fastest early childbearing decline occurred among rural women, whereas no clear pattern was observed for wealth groups. Decreasing estimates from oldest to youngest generations were found among Afro-descendants and non-Afro-descendant and non-indigenous groups, but results were mixed for indigenous people. All nine countries with data for AFR presented reductions over time (–0·7 to –6·5 births per 1000 women per year), with the steepest declines observed in Ecuador, Guyana, Guatemala, and the Dominican Republic. In general, adolescents in rural areas and the poorest adolescents had the largest reductions in AFR. If current trends persist, by 2030 most countries will present AFR values ranging between 45 and 89 births per 1000 women, with notable wealth-related inequalities.

**Interpretation:**

Our results indicate a reduction in AFR in Latin American and Caribbean countries that was not necessarily accompanied by a decrease in early childbearing overall. Large inequalities both between countries and within countries were observed, with no clear reduction over time. Understanding trends in adolescent childbearing and its determinants is essential for planning and designing programmes to ensure the desired reductions in rates and gaps across population subgroups.

**Funding:**

PAHO, Bill & Melinda Gates Foundation, and Wellcome Trust.

**Translations:**

For the Spanish and Portuguese translations of the abstract see Supplementary Materials section.

## Introduction

Adolescent girls aged 15–19 years from low-income and middle-income countries are estimated to have approximately 21 million pregnancies every year, half of which are unintended.^1^ Adolescent childbearing is associated with poor maternal and child outcomes, and complications during pregnancy and childbirth are among the leading cause of death for adolescent girls.^2^, ^3^ It is also associated with undesirable social and economic consequences such as increased school dropout rates, reduction in education years, and worse employment status and income level in the future, resulting in an enormous societal burden.^1^, ^4^ Additionally, it contributes to perpetuating the cycle of intergenerational poverty, especially for women who live in poor, low-education, and rural communities.^1^, ^5^ Recognising these severe consequences, Sustainable Development Goal (SDG) 3.7 asks countries to ensure universal access to sexual and reproductive health care services by 2030, including family planning, information and education, and the integration of reproductive health into national strategies and programmes.^6^

Adolescent childbearing is common in many countries with remarkable regional differences. Despite showing a reduction since 2000, the region of Latin America and the Caribbean (LAC) presented the second-highest adolescent fertility rate (AFR) in the world in 2020, with 61 births per 1000 women aged between 15 and 19 years.^1^, ^7^ A modelling analysis of adolescent fertility in 32 countries in the region of the Americas found that the studied countries had reductions in AFRs, but the speed of decline was different across study geographies and between the two time periods modelled (1960–89 and 1990–2019), with a slower reduction in the most recent years.^8^

**Research in context**

**Evidence before this study**

Latin America and the Caribbean (LAC) have undergone rapid demographic and social changes in the past decades, with major implications for adolescent health. Despite the decline in adolescent fertility rates (AFRs), the region presented the second-highest estimate of AFR in 2020, and reached the third-highest position globally in the incidence of motherhood among adolescent girls. It is also known that socioeconomic inequalities within countries and across countries are high, and these associated factors are decisive for adolescent childbearing. We searched PubMed combining the terms related to adolescent motherhood (eg, ‘adolescent’ or ‘young adult’ and ‘pregnancy’ or ‘reproductive health’) and LAC country names in November 2021, with an updated search in October 2022. There was no language restriction. We found few studies describing trends in either adolescent fertility or early childbearing indicators. Also, those studies covering multiple countries often worked with just a few countries, or used country-level estimates that do not allow subgroup analyses. Characteristics such as poverty and rural residence have been found to be associated with teenage pregnancy and early motherhood; however, to our knowledge, there has been no recent comprehensive report of trends in adolescent childbearing, comparing early childbearing and fertility, which also includes the assessment of inequities across population subgroups in multiple countries in the region.

**Added value of this study**

In this study, we expanded the data used in previous works and used a novel approach to estimate adolescent childbearing over time in LAC countries. In addition to time trends in adolescent fertility rates (livebirths per 1000 women aged 15–19 years), we calculated early childbearing as the proportion of women having a first livebirth before 18 years of age. For this later indicator, we were able to analyse the trends across generations of women, considering their age at the time of the survey. We included Demographic and Health Surveys, Multiple Indicator Cluster Surveys, and other national surveys, which allowed us to analyse data from 21 of the 33 LAC countries. The analysis of survey microdata also permitted us to explore trends in inequities considering important population stratifiers, namely wealth, area of residence, and ethnicity. Therefore, this study provides updated estimates of levels and trends of adolescent fertility and early childbearing, based on nationally representative survey data, along with analysis of inequalities among population subgroups, which are important inputs to epidemiological research and design. Our results indicate that adolescent childbearing continues to be a major issue in the LAC region, although there has been a reduction in the average number of livebirths among adolescents, along with a decrease in the proportion of adolescents who become mothers. Nevertheless, some countries presented a stable or increased prevalence of early childbearing. Adolescent girls from families in the lower wealth groups, living in rural areas, and from indigenous and Afro-descendant groups were disproportionately affected by adolescent motherhood, and the magnitude of the inequalities in the changes varied across setting.

**Implications of all the available evidence**

The findings of this study extend our understanding of country-specific changes in adolescent childbearing over time and the persistent inequality between population subgroups. The analysis highlights the need for context-specific measurements, evaluation, and policy. Monitoring different indicators of adolescent childbearing is an important approach for addressing sexual and reproductive health and rights of adolescent girls in the LAC region, given the complex dynamic of this issue. These results must be analysed together with other critical elements that impact adolescent health, such as safe abortion, gender norms, and high-quality and timely sexual and reproductive health services. This needs to happen alongside prioritising the needs of vulnerable and neglected populations.

The proportion of adolescent girls bearing a child has shown a modest decline in the past 50 years, placing LAC in the third position among all world regions.^1^, ^7^ Data from household surveys indicated that only three of nine LAC countries analysed in one study presented a decreasing trend in adolescent motherhood (measured as women aged between 15 and 19 years who either had a livebirth or were pregnant at the time of the survey) between 1990 and 2018.^9^

Poverty and lack of economic opportunities are associated with teenage pregnancy and early motherhood, but few studies on adolescent childbearing address within-country inequalities by comparing population subgroups. Those that have addressed subgroups found important inequalities within countries.^9^, ^10^, ^11^ A study analysed data from national surveys from five LAC countries (Bolivia, Colombia, Dominican Republic, Haiti, and Peru), carried out between 1990 and 2006, and did not observe reductions in adolescent childbearing, measured as the proportion of women aged 20–24 years who have had a child before age 20 years, except in Haiti; also, there was a higher proportion of births in poorer women and women living in rural areas compared with women from urban areas or wealthier backgrounds, and in some countries, these differentials increased over time.^10^

Despite the accumulated knowledge, key gaps remain in the understanding of regional trends of adolescent motherhood across population subgroups. Use of aggregate data in ecological studies does not allow stratification by socioeconomic and demographic characteristics. The number of countries included in the analyses is usually small, hindering a better picture of the region, and when individual data are available, subgroups are ususally restricted to age and wealth. In this work, we aimed to update and expand on the previous literature by using nationally representative surveys carried out in multiple LAC countries to study trends and inequalities in adolescent childbearing. We assessed whether there was a general reduction in the proportion of women having a first birth in adolescence across generations and in AFRs and described how changes varied across population subgroups defined by wealth, area of residence, and ethnicity.

## Methods

### Study design and data sources

In this population-based study, we used data from nationally representative household surveys conducted in LAC countries as used by the UN. We used Multiple Indicator Cluster Surveys, Demographic and Health Surveys (DHS), and other national surveys with similar sampling designs that allowed comparable indicators to be estimated, namely, the 2016 Bolivian *Encuesta de Demografía y Salud* (EDSA) and the 2012 and 2018 Ecuadorian *Encuesta Nacional de Salud y Nutrición* (ENSANUT).^12^, ^13^, ^14^, ^15^, ^16^

### Indicators

We addressed trends in early childbearing across generations as the proportion of women in different birth cohorts who had their first livebirth before 18 years of age, following the definition used by UNICEF and other international organisations. Early childbearing is presented in the Multiple Indicator Cluster Surveys reports under the “Thrive—reproductive and maternal health” section (indicator TM 2) and is defined as the proportion of women aged 20–24 years who have had a livebirth before age 18 years. Multiple Indicator Cluster Surveys and DHS reports also present this indicator for other age groups, including older women.

We looked at trends on early childbearing by splitting the women in the sample into six 5-year groups based on age at survey completion: 20–24 years, 25–29 years, 30–34 years, 35–39 years, 40–44 years, and 45–49 years. We refer to these groups as generations for the sake of simplicity. The aim of this analysis by age groups was to produce estimates of early childbearing for the current and past generations of women. For this set of analyses, we included the latest survey of any type of each LAC country conducted since 2010. We did not present estimates for these age groups when the unweighted denominator included 25 observations or fewer.^17^

The second indicator used was AFR, defined as the number of livebirths that occurred in women aged 15–19 years at the time of birth. It was estimated based on a synthetic cohort approach and expresses the number of livebirths per 1000 women-years in this age group in the 5 years preceding the survey.^17^ For this indicator, countries with at least two surveys, with the latest conducted in 2010 onwards, were included. All the available surveys for these countries were used in the trend analyses**.** Following the DHS precision criteria, we omitted estimates based on less than 125 women-years of exposure.^17^

### Statistical analysis

For the two indicators, we calculated estimates and the corresponding SEs and 95% CIs at the country level (national estimates), and by wealth groups, area of residence, and ethnicity, considering the complex sampling design of the surveys, accounting for clustering, sample weights, and strata using Stata's *svy* prefix with default options as recommended by the survey guidelines. We explored the linear association between early childbearing among women aged 20–24 years at the time of the survey (most recent generation) and AFR estimates in the latest survey at the country level using Pearson's correlation.

The asset index used to create the wealth categories was provided with the survey data and it was generated using the first factors produced by a principal components analysis based on variables related to household assets, building materials of the dwelling, and availability of utilities, such as electricity and water.^18^ The index creation was based on separate factor scores for households in urban and rural areas, using indicators relevant to each of them as per country and survey definition, such as size of landholdings, and number of farm animals for rural areas and publicly provided services (electricity, piped water, sewers) for urban areas. A common score was derived through a regression-based scaling procedure.^18^ Subsequently, households were categorised into five equally sized groups (wealth quintiles), each one representing 20% of the sample.^18^, ^19^ In this analysis, we compared women from the bottom 40% of households (quintiles 1–2) with the top 60% (quintiles 3–5), as previously employed in the literature of health inequalities.^20^, ^21^ Area of residence was defined as urban or rural according to the country's specific delimitation at the time of the survey. Ethnicity was defined as indigenous, Afro-descendant, or other non-indigenous and non-Afro-descendant groups, as employed in previous studies.^22^, ^23^ Other non-indigenous and non-Afro-descendant groups included women of European descent, those of mixed ancestry, and those who did not report a specific ethnic affiliation. The classification was based upon the country-specific context and might refer to women's or household head's information, depending on the data source.^22^, ^23^

For early childbearing, trends were estimated using the six age groups created, each representing one time period preceding the survey, interpreted as generations. An ordinal variable ranging from 1 to 6 was the independent variable, where each category represents an age group, with group 1 being older women (45–49 years old) and group 6 being younger women (20–24 years old). With the proportion of women who had a child before 18 years of age as the dependent variable, country-specific variance-weighted least squares (VWLS) regression models were used to estimate the average absolute change (AAC) across generations (from 45–49 years to 20–24 years) at the national level, and by categories of stratifiers (wealth, area of residence, and ethnicity). The coefficient obtained from the models represents the AAC for the country or subgroup over generations—from the oldest to the youngest—measured in percentage points, and differences between them were assessed based on the AAC (95% CI). Therefore, negative AAC values indicate a reduction in early childbearing across generations in percentage points, while positive values reflect an increase in early childbearing.

Trends in AFR were estimated for countries that had conducted more than one survey, the latest being done since 2010. The same regression models were used to derive the annual AAC for this indicator, using the survey year as a predictor and the AFR as the outcome. Annual AACs were calculated at the national level and by categories of wealth, area, and ethnicity. For this outcome, negative values indicate an annual reduction in AFR—expressed as the number of livebirths per 1000 adolescents per year—while positive values indicate an increase in AFR. To check whether the slopes for the wealth groups were statistically different from each other, we assessed interactions between wealth categories and survey year in our VWLS models by adding product terms between the two indicator variables. Significant interaction indicates that the speed of change over time was different for the two wealth groups. Additionally, using linear regression models and based on the annual AAC values and the adolescent fertility estimate for the most recent survey from each country, we estimated what would be the country-specific AFR by 2030, assuming that the current progression is maintained.

For all analysis, statistical significance was assessed considering an α level of 0·05. All analyses were carried out with Stata version 17.

### Role of the funding source

The funder of the study had no role in study design, data collection, data analysis, data interpretation, or writing of the report.

## Results

We included 21 countries, with the most recent survey conducted from 2010 onwards, to analyse early childbearing (15 Multiple Indicator Cluster Surveys, four DHS, and two of the other national surveys); survey years ranged from 2010 to 2020. For this indicator, 241 333 women aged 20–49 years were included, representing 1 395 110 individuals (weighted estimate). For analysis of AFR, 51 surveys (five Multiple Indicator Cluster Surveys, 43 DHS, and three other surveys; 1994 –2020) from nine countries were included, representing 858 594 women-years. The number of surveys by country ranged from two from Ecuador to 18 from Peru.

Based on the most recent survey available for LAC countries from 2010 onwards, the proportion of women aged 20–24 years having their first birth before age 18 years ranged from 6·3% (95% CI 4·5–8·8) in Trinidad and Tobago to 25·8% (24·0–27·6) in Honduras (median 14**·**1%, IQR 11·4–18·2), whereas estimates for AFR varied from 54 births per 1000 adolescents (95% CI 50–58) in Peru to 97 (92–103) in Honduras (median 74, IQR 70·3–80·9; Table 1, Table 2). We identified a Pearson's correlation coefficient of *r*=0·83 (p=0·0010) for the linear association between early childbearing among women aged 20–24 years and AFR at the country level, indicating that countries with a higher proportion of women having a first child before 18 years of age also have a higher AFR (appendix 3 p 15).

**Table 1:** Estimates of early childbearing based on the most recent household survey from Latin American and Caribbean countries

	**Women who had their first birth before the age of 18, % (95% CI)**						**AAC (95% CI)**
	45–49 years	40–44 years	35–39 years	30–34 years	25–29 years	20–24 years	
Argentina	15·3% (12·5–18·7)	13·6% (11·0–16·8)	16·5% (13·7–19·7)	12·7% (10·5–15·1)	15·0% (12·5–17·8)	14·1% (11·9–16·6)	−0·2 (−0·8 to 0·5)
Barbados	14·4% (9·9–20·6)	11·1% (7·1–17·0)	13·0% (9·0–18·3)	7·3% (4·4–11·9)	8·9% (5·8–13·5)	6·7% (3·9–11·5)	−1·4 (−2·5 to −0·4)
Belize	23·7% (18·7–29·6)	24·1% (19·4–29·5)	24·3% (19·7–29·7)	21·0% (17·6–24·8)	19·7% (16·4–23·5)	17·3% (14·5–20·5)	−1·5 (−2·5 to −0·5)
Bolivia	18·6% (15·4–22·4)	15·5% (13·2–18·3)	15·6% (13·5–18·0)	17·9% (15·9–20·2)	15·8% (13·8–18·0)	17·1% (14·9–19·5)	0·0 (−0·6 to 0·6)
Colombia	14·1% (12·7–15·6)	16·6% (15·0–18·2)	19·4% (17·5–21·5)	21·8% (20·0–23·8)	20·0% (18·2–22·0)	19·5% (17·9–21·1)	1·2 (0·8 to 1·5)
Costa Rica	13·6% (11·0–16·8)	20·6% (16·9–25·0)	20·3% (17·0–24·0)	15·6% (12·6–19·3)	13·9% (11·5–16·7)	13·1% (10·8–15·7)	−0·6 (−1·3 to 0·0)
Cuba	16·8% (13·7–20·5)	15·4% (11·4–20·4)	11·3% (8·2–15·4)	13·3% (10·4–16·7)	7·9% (6·0–10·3)	10·3% (8·0–13·2)	−1·5 (−2·3 to −0·8)
Dominican Republic	20·8% (18·7–23·1)	22·8% (20·8–24·9)	27·6% (25·4–29·9)	24·9% (22·6–27·5)	20·4% (18·6–22·3)	20·4% (18·7–22·3)	−0·5 (−0·9 to 0·0)
Ecuador	14·6% (12·9–16·5)	18·4% (16·3–20·7)	21·0% (19·0–23·0)	21·8% (20·1–23·7)	22·1% (20·2–24·0)	10·9% (9·7–12·2)	−0·9 (−1·2 to −0·5)
El Salvador	23·9% (21·1–27·0)	23·0% (20·3–26·0)	24·1% (21·4–26·9)	25·3% (22·7–28·2)	19·1% (17·0–21·3)	18·2% (16·4–20·1)	−1·3 (−1·9 to −0·8)
Guatemala	24·0% (21·9–26·4)	23·0% (21·1–25·0)	23·8% (22·2–25·6)	24·3% (22·8–25·9)	21·0% (19·5–22·6)	20·2% (18·7–21·7)	−0·8 (−1·2 to −0·4)
Guyana	23·1% (19·2–27·6)	20·5% (16·2–25·7)	21·8% (17·8–26·4)	21·9% (17·8–26·5)	14·8% (11·7–18·6)	13·5% (10·3–17·4)	−2·0 (−2·9 to −1·1)
Haiti	15·6% (13·2–18·3)	17·2% (15·0–19·7)	15·8% (13·8–18·1)	16·2% (14·4–18·2)	13·9% (12·0–16·0)	13·6% (12·1–15·3)	−0·6 (−1·1 to −0·1)
Honduras	21·4% (19·3–23·7)	25·7% (23·6–27·9)	27·4% (25·3–29·6)	25·8% (23·9–27·9)	24·0% (22·2–26·0)	25·8% (24·0–27·6)	0·4 (−0·1 to 0·9)
Jamaica	27·6% (23·3–32·4)	25·6% (21·6–30·1)	21·9% (18·8–25·4)	23·7% (19·7–28·2)	19·5% (15·9–23·7)	14·9% (12·2–18·0)	−2·4 (−3·3 to −1·6)
Mexico	13·0% (10·0–16·6)	17·2% (14·0–20·8)	13·9% (11·7–16·5)	18·7% (15·2–22·9)	16·6% (13·5–20·2)	20·5% (17·8–23·5)	1·3 (0·5 to 2·0)
Paraguay	14·0% (10·8–17·8)	16·8% (13·4–20·8)	19·6% (16·2–23·5)	16·8% (13·8–20·2)	15·3% (12·7–18·2)	14·8% (12·5–17·4)	−0·2 (−0·9 to 0·5)
Peru	13·4% (11·7–15·3)	13·9% (12·2–15·7)	14·9% (13·5–16·5)	14·3% (13·0–15·7)	15·0% (13·6–16·5)	11·4% (10·2–12·7)	−0·4 (−0·7 to −0·0)
Saint Lucia	23·5% (17·1–31·5)	20·6% (15·0–27·5)	15·0% (9·5–22·8)	18·1% (12·3–25·9)	12·1% (7·2–19·6)	9·3% (6·0–14·0)	−2·7 (−4·0 to −1·4)
Suriname	18·8% (15·4–22·6)	15·0% (11·9–18·7)	15·7% (12·1–20·3)	16·5% (13·8–19·6)	13·7% (11·1–16·8)	13·2% (11·0–15·8)	−0·9 (−1·6 to −0·2)
Trinidad and Tobago	11·9% (9·4–15·0)	10·1% (7·7–13·2)	11·2% (8·6–14·4)	7·7% (5·6–10·4)	7·1% (5·2–9·6)	6·3% (4·5–8·8)	−1·1 (−1·7 to −0·6)

AAC=average absolute change.

**Table 2:** Estimates of AFR and corresponding annual AAC for Latin American and Caribbean countries by survey year

	**National**	**Wealth**		**Area of residence**		**Ethnicity**		
		Bottom 40%	Top 60%	Rural	Urban	Indigenous	Afro-descendant	Non-Indigenous and non-Afro-descendant
**Bolivia**								
1994	96 (88 to 105)	138 (119 to 156)	78 (70 to 87)	124 (106 to 142)	82 (73 to 91)	NA	NA	NA
1998	88 (79 to 96)	151 (135 to 166)	65 (56 to 73)	141 (124 to 158)	71 (62 to 80)	NA	NA	NA
2003	97 (90 to 104)	163 (149 to 176)	73 (66 to 79)	145 (132 to 159)	78 (71 to 86)	NA	NA	NA
2008	89 (82 to 95)	146 (132 to 159)	64 (58 to 70)	135 (122 to 148)	68 (61 to 75)	NA	NA	NA
2016	74 (67 to 82)	118 (102 to 134)	51 (45 to 57)	122 (101 to 143)	58 (52 to 65)	NA	NA	NA
Annual AAC	−0·9 (−1·3 to −0·4)	−1·1 (−2·1 to −0·2)	−1·1 (−1·5 to −0·7)	−0·2 (−1·3 to 0·9)	−1·0 (−1·4 to −0·6)	NA	NA	NA
**Colombia**								
1995	92 (85 to 99)	149 (135 to 164)	62 (55 to 69)	142 (124 to 160)	75 (68 to 83)	NA	NA	NA
2000	85 (78 to 92)	144 (131 to 157)	56 (50 to 63)	136 (119 to 152)	72 (65 to 79)	NA	NA	NA
2005	92 (87 to 97)	140 (132 to 148)	65 (59 to 70)	138 (128 to 148)	79 (73 to 84)	NA	NA	NA
2010	85 (81 to 89)	130 (123 to 137)	58 (54 to 62)	128 (119 to 138)	74 (70 to 78)	119 (103 to 135)	99 (88 to 110)	82 (78 to 86)
2015	77 (72 to 82)	121 (113 to 128)	46 (41 to 51)	125 (115 to 136)	64 (58 to 69)	116 (96 to 136)	93 (79 to 107)	73 (68 to 78)
Annual AAC	−0·7 (−1·0 to −0·3)	−1·5 (−2·2 to −0·9)	−0·7 (−1·0 to −0·3)	−0·9 (−1·8 to −0·0)	−0·5 (−0·9 to −0·1)	−0·6 (−5·7 to 4·5)	−1·2 (−4·7 to 2·4)	−1·7 (−3·0 to −0·4)
**Dominican Republic**								
1996	115 (104 to 125)	192 (174 to 210)	77 (67 to 87)	166 (148 to 184)	89 (77 to 101)	NA	NA	NA
1999	101 (72 to 130)	162 (121 to 203)	68 (41 to 95)	134 (105 to 163)	83 (40 to 126)	NA	NA	NA
2002	118 (110 to 125)	184 (173 to 194)	81 (73 to 89)	145 (133 to 158)	105 (96 to 114)	NA	NA	NA
2007	98 (92 to 104)	157 (147 to 166)	65 (59 to 71)	129 (119 to 139)	86 (79 to 93)	NA	NA	NA
2013	90 (81 to 99)	138 (125 to 152)	59 (49 to 69)	102 (84 to 120)	86 (75 to 96)	NA	NA	NA
2014	91 (86 to 96)	145 (136 to 154)	60 (55 to 65)	113 (104 to 123)	85 (79 to 91)	NA	NA	NA
2019	81 (75 to 87)	132 (121 to 143)	47 (41 to 53)	112 (98 to 126)	71 (65 to 78)	NA	NA	NA
Annual AAC	−1·7 (−2·1 to −1·3)	−2·8 (−3·4 to −2·1)	−1·4 (−1·9 to −1·0)	−2·4 (−3·1 to −1·6)	−1·1 (−1·6 to −0·6)	NA	NA	NA
**Ecuador**								
2012	112 (100 to 124)	146 (128 to 164)	91 (80 to 102)	114 (101 to 128)	111 (93 to 129)	146 (127 to 165)	126 (95 to 158)	109 (97 to 121)
2018	73 (69 to 78)	101 (95 to 108)	53 (47 to 58)	96 (89 to 103)	63 (58 to 69)	85 (72 to 99)	94 (72 to 117)	70 (65 to 75)
Annual AAC	−6·5 (−8·7 to −4·3)	−7·4 (−10·6 to −4·2)	−6·4 (−8·4 to −4·4)	−3·1 (−5·7 to −0·5)	−8·0 (−11·1 to −4·8)	−10·1 (−14·1 to −6·1)	−5·4 (−11·8 to 1·1)	−6·5 (−8·6 to −4·3)
**Guatemala**								
1995	127 (116 to 138)	188 (171 to 205)	97 (84 to 109)	152 (139 to 164)	95 (76 to 114)	NA	NA	NA
1998	123 (109 to 137)	181 (164 to 198)	93 (77 to 108)	146 (127 to 164)	95 (75 to 115)	NA	NA	NA
2014	94 (88 to 99)	131 (122 to 140)	70 (65 to 76)	116 (108 to 123)	64 (58 to 71)	NA	NA	NA
Annual AAC	−1·8 (−2·4 to −1·2)	−3·1 (−3·9 to −2·2)	−1·4 (−2·0 to −0·8)	−1·9 (−2·6 to −1·2)	−1·7 (−2·6 to −0·9)	NA	NA	NA
**Guyana**								
2009	97 (78 to 115)	160 (122 to 198)	58 (46 to 71)	116 (91 to 140)	49 (38 to 61)	214 (136 to 293)	64 (47 to 82)	90 (73 to 108)
2014	77 (66 to 88)	140 (119 to 160)	38 (30 to 46)	85 (71 to 98)	57 (41 to 72)	158 (124 to 192)	60 (44 to 76)	76 (62 to 90)
2019	70 (60 to 81)	116 (98 to 134)	44 (33 to 55)	72 (59 to 85)	65 (46 to 85)	147 (115 to 179)	52 (38 to 67)	70 (56 to 83)
Annual AAC	−2·3 (−4·3 to −0·4)	−4·4 (−8·2 to −0·7)	−1·3 (−2·9 to 0·4)	−3·9 (−6·4 to −1·4)	1·6 (−0·6 to 3·8)	−4·8 (−11·8 to 2·2)	−1·2 (−3·5 to 1·0)	−2·0 (−4·2 to 0·2)
**Haiti**								
1994	79 (68 to 89)	111 (90 to 131)	66 (54 to 78)	93 (79 to 107)	63 (46 to 79)	NA	NA	NA
2000	80 (70 to 90)	96 (76 to 116)	73 (62 to 84)	100 (83 to 116)	61 (46 to 76)	NA	NA	NA
2005	69 (62 to 75)	92 (79 to 104)	59 (52 to 67)	86 (77 to 95)	51 (43 to 60)	NA	NA	NA
2012	65 (59 to 72)	88 (78 to 99)	55 (48 to 62)	76 (68 to 84)	54 (45 to 63)	NA	NA	NA
2016	59 (54 to 65)	87 (77 to 97)	46 (40 to 52)	73 (66 to 80)	43 (35 to 51)	NA	NA	NA
Annual AAC	−0·9 (−1·4 to −0·5)	−0·8 (−1·7 to −0·0)	−1·1 (−1·6 to −0·6)	−1·1 (−1·8 to −0·5)	−0·8 (−1·5 to −0·2)	NA	NA	NA
**Honduras**								
2005	108 (102 to 113)	150 (142 to 159)	88 (82 to 95)	137 (130 to 145)	84 (76 to 91)	NA	NA	NA
2011	99 (94 to 104)	145 (137 to 153)	75 (70 to 81)	120 (112 to 127)	82 (75 to 89)	83 (71 to 95)	72 (48 to 96)	103 (97 to 108)
2019	97 (92 to 103)	137 (128 to 146)	75 (69 to 81)	117 (110 to 124)	76 (68 to 84)	96 (81 to 110)	94 (36 to 153)	99 (93 to 105)
Annual AAC	−0·7 (−1·3 to −0·2)	−1·0 (−1·9 to −0·1)	−0·9 (−1·5 to −0·2)	−1·4 (−2·1 to −0·6)	−0·5 (−1·3 to 0·2)	1·6 (−0·8 to 4·0)	2·8 (−5·1 to 10·7)	−0·5 (−1·5 to 0·6)
**Peru**								
1996	77 (72 to 82)	144 (129 to 160)	46 (41 to 52)	141 (130 to 152)	56 (51 to 61)	NA	NA	NA
2000	70 (65 to 75)	132 (121 to 144)	42 (37 to 47)	124 (115 to 133)	49 (44 to 54)	NA	NA	NA
2004	59 (50 to 68)	109 (87 to 130)	41 (32 to 50)	101 (83 to 120)	45 (35 to 54)	NA	NA	NA
2005	63 (51 to 75)	118 (81 to 154)	40 (29 to 51)	109 (85 to 134)	43 (32 to 54)	NA	NA	NA
2006	62 (53 to 71)	125 (93 to 157)	38 (28 to 48)	121 (101 to 142)	42 (34 to 50)	NA	NA	NA
2007	64 (54 to 73)	130 (104 to 156)	45 (33 to 56)	113 (96 to 130)	46 (36 to 56)	NA	NA	NA
2008	65 (57 to 73)	128 (102 to 153)	47 (40 to 54)	115 (101 to 129)	49 (41 to 56)	NA	NA	NA
2009	69 (64 to 74)	120 (107 to 134)	43 (36 to 50)	121 (110 to 133)	52 (47 to 57)	NA	NA	NA
2010	72 (66 to 77)	115 (101 to 129)	49 (41 to 57)	117 (106 to 127)	57 (50 to 63)	NA	NA	NA
2011	62 (57 to 67)	105 (92 to 118)	40 (33 to 46)	105 (96 to 115)	49 (44 to 55)	NA	NA	NA
2012	67 (61 to 72)	114 (100 to 128)	42 (35 to 48)	114 (104 to 124)	51 (46 to 57)	NA	NA	NA
2013	68 (61 to 74)	106 (92 to 119)	46 (39 to 53)	113 (102 to 124)	55 (48 to 62)	NA	NA	NA
2014	67 (62 to 72)	104 (91 to 117)	46 (40 to 52)	115 (105 to 125)	54 (49 to 59)	NA	NA	NA
2015	64 (60 to 68)	108 (99 to 118)	39 (35 to 43)	119 (110 to 127)	51 (47 to 54)	NA	NA	NA
2016	63 (59 to 67)	108 (99 to 117)	38 (34 to 42)	122 (113 to 132)	48 (44 to 52)	NA	NA	NA
2017	64 (59 to 69)	103 (93 to 113)	39 (34 to 45)	119 (110 to 129)	51 (46 to 56)	NA	NA	NA
2018	53 (49 to 56)	94 (85 to 103)	29 (25 to 33)	114 (104 to 123)	40 (37 to 44)	NA	NA	NA
2019	54 (50 to 58)	96 (87 to 105)	29 (26 to 33)	113 (104 to 122)	42 (39 to 46)	NA	NA	NA
Annual AAC	−0·8 (−1·0 to −0·6)	−1·9 (−2·4 to −1·4)	−0·6 (−0·8 to −0·4)	−0·6 (−1·0 to −0·2)	−0·4 (−0·6 to −0·2)	NA	NA	NA

Data are AFR (95% CI) or AAC (95% CI). Estimates based on less than 125 women-years of exposure have been omitted. Negative annual AAC values indicate a reduction in the number of births per 1000 adolescent girls each year whereas positive values indicate an annual increase in the figures. Not all surveys used for national, wealth, and area estimates have collected information on ethnicity and are marked with NA. AFR=adolescent fertility rate (livebirths per 1000 women aged 15–19 years in the 5 years preceding the survey). AAC=average absolute change. NA=not applicable.

Among the 21 LAC countries for which we had a survey since 2010 and information on a woman's age at first birth, we observed a reduction in early childbearing across generations, represented by a negative AAC, in 13 of them: Saint Lucia, Jamaica, Guyana, Cuba, Belize, Barbados, El Salvador, Trinidad and Tobago, Suriname, Ecuador, Guatemala, Haiti, and Peru (countries listed from largest decrease to smallest decrease in adolescent childbearing; table 1, appendix 3 pp 5,17). These reductions indicate that having a first birth in adolescence was less frequent among younger generations of women than among older ones. The magnitudes of reduction, where observed, were small, with Saint Lucia showing the maximum percentage point change (–2·7 [95% CI –4·0 to –1·4]) with each generation change. Conversely, we noted increases in early childbearing in Mexico and Colombia (appendix 3 p 5).

**Figure 1:**
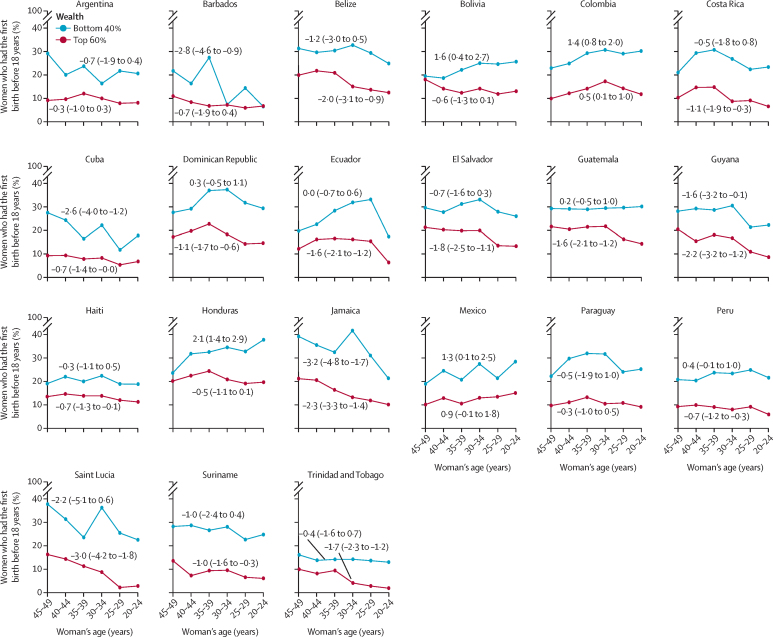
Trends in early childbearing by wealth groups in Latin American and Caribbean countries across generations Early childbearing indicates the proportion of women who had their first birth before the age of 18 years. Negative numbers (95% CI) indicate a reduction and positive numbers (95% CI) indicate an increase across the generations. Wealth groups are bottom 40% and top 60% of the wealth index distribution. Numbers indicate average absolute change.

In all countries and age groups, early childbearing was higher for the 40% in the bottom of the wealth distribution when compared with the top 60% (figure 1, table 2). Four countries (Jamaica, Barbados, Cuba, and Guyana) had a significant decline in early childbearing across generations among the poorest wealth group, represented by negative AAC values (figure 1). Conversely, in 14 countries women in the wealthiest group (top 60%) had a decline in early childbearing.

When comparing the changes between the wealth categories within each country we found that among the 14 countries where both groups had declines across generations, in six countries the magnitude of reduction was higher among the poorest groups, which would indicate a faster decline; however, as mentioned before, in most of them the changes were not significant among the poorest groups (appendix 3 pp 6–7).

**Figure 2:**
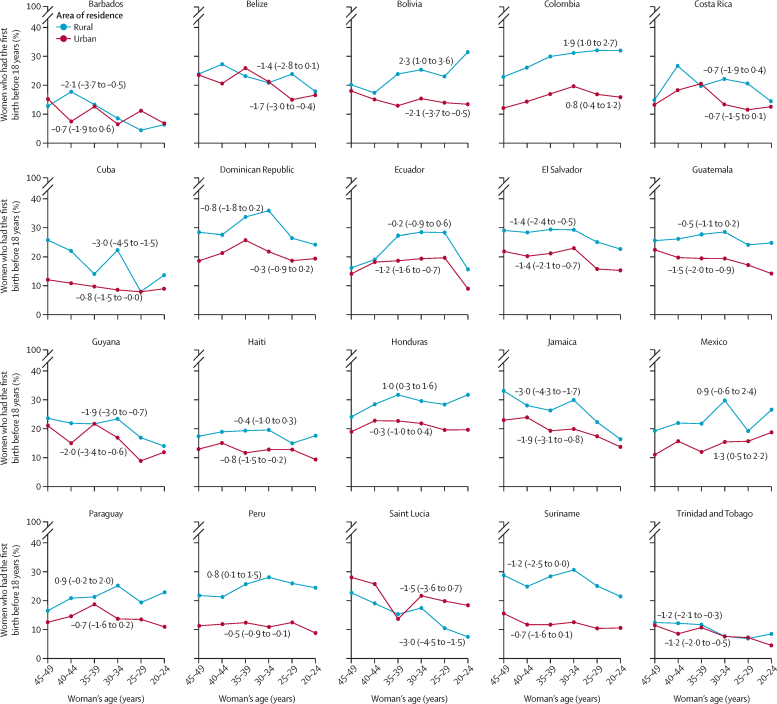
Trends in early childbearing by urban versus rural residence in Latin American and Caribbean countries across generations Early childbearing indicates proportion of women who had their first birth before the age of 18 years. Negative numbers (95% CI) indicate a reduction and positive numbers (95% CI) indicate an increase across the generations. Numbers indicate average absolute change.

Information on the area of residence was available for 20 countries, and in ten of them, women from urban areas showed a significant decline in early childbearing across generations. Among women from the rural areas, seven countries presented declines (Barbados, Cuba, El Salvador, Guyana, Jamaica, Saint Lucia, Trinidad and Tobago) and four presented increases across generations (Bolivia, Colombia, Honduras, and Peru, figure 2; appendix 3 pp 8–9).

**Figure 3:**
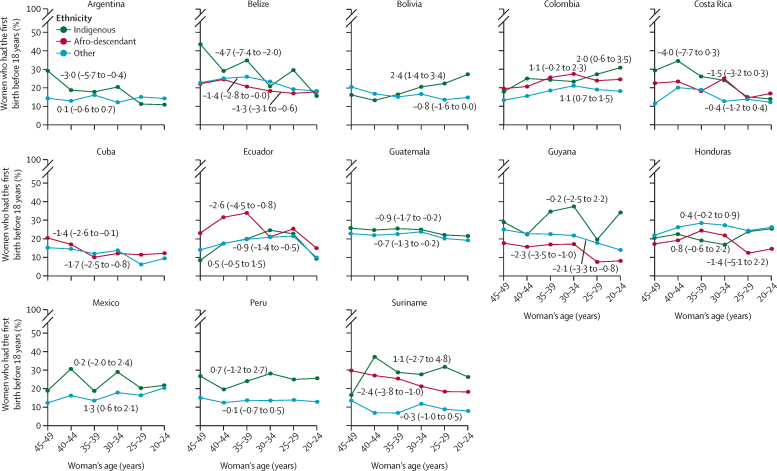
Trends in early childbearing by ethnicity in Latin American and Caribbean countries across generations Early childbearing indicates the proportion of women who had their first birth before the age of 18 years. Negative numbers (95% CI) indicate a reduction and positive numbers (95% CI) indicate an increase across the generations. Numbers indicate average absolute change.

Given data availability and sample size limitations, we were only able to analyse adolescent childbearing by ethnic groups for 13 countries. Overall, negative AACs, indicating a decrease in early childbearing across generations, were observed in the group of non-indigenous and non-Afro-descendant women (significant in five countries: Belize, Cuba, Ecuador, Guatemala, and Guyana) and for the group of Afro-descendants (reported for eight countries; significant in Cuba, Ecuador, Guyana, and Suriname). Although mixed results were observed for indigenous peoples, significant decreases were observed in four countries: Argentina, Belize, Costa Rica, and Guatemala (figure 3**;** appendix 3 pp 10–11).

For the analyses of AFR, we identified nine eligible countries with the number of surveys per country varying from two in Ecuador to 18 in Peru (table 2). At the national level, we observed a pattern of decreasing rates over time for all countries studied, represented by the negative annual AAC estimates, all of which were statistically significant. Five countries presented an average reduction of less than one birth per 1000 women per year (Bolivia, Colombia, Haiti, Honduras, and Peru). Three countries presented values between one and three (Dominican Republic, Guatemala, and Guyana). The greatest reduction was observed in Ecuador, with 6·5 fewer births per 1000 women per year (95% CI –8·7 to –4·3); however, the country had only two surveys available (appendix 3 p 17).

We observed systematic differences in fertility, with the poorest groups, when compared to the wealthiest 60%, always presenting the highest rates of reduction. Both wealth groups presented decreasing AFR over time, with Guyana being the only country with a non-significant AAC, which was observed among women from the top 60% wealth group (appendix 3 p 18). Based on the statistically significant interaction test between survey year and wealth groups, we observed that in four countries (Colombia, Dominican Republic, Guatemala, and Peru; appendix 3 pp 12–13) the decline in AFR was faster among poorest adolescents than in those in the top wealth groups. In the other five countries, we found no evidence of a different annual AAC between wealth groups.

According to the predictions based on observed trends, and assuming that no additional interventions are in place, AFR would be as low as 45 in Guyana and as high as 89 births per 1000 women in Honduras in 2030 (appendix 3 p 14). Nevertheless, when looking at the estimates by wealth, adolescents in the lower wealth bracket are not expected to experience the same birth rate as their wealthier peers, even in those countries where the AFR decline was faster among the poorest. By 2030, according to this forecast, adolescents in the poorest group will have AFRs that are 1·7 to 3·4 times higher than adolescents in the upper wealth group.

All countries presented negative estimates for fertility annual AAC in both rural and urban areas (table 2, appendix 3 p 19), indicating a decline in fertility over time, except for urban areas in Guyana. The changes were statistically different from zero in both urban and rural areas in Colombia, the Dominican Republic, Ecuador, Guatemala, Haiti, and Peru. We observed a significant decrease in urban areas only in Bolivia, and in rural areas only in Guyana and Honduras.

Information on ethnicity in multiple surveys was available for four countries only. Significant reductions in AFR over time were observed for the non-indigenous and non-Afro-descendant group in Colombia and Ecuador (table 2, appendix 3 p 20). In Ecuador, indigenous adolescents presented an extreme annual AAC reduction of 10·1 births per 1000 (95% CI –14·1 to –6·1). Caution is needed with estimates for Ecuador, as only two surveys 6 years apart were available.

## Discussion

Our analyses suggest that there has been progress, mostly in reducing AFR, in the LAC region. However, the region still has the second highest AFR estimates among world regions.^1^ For early childbearing, only 13 of 21 countries (62%) presented a statistically significant decrease. As expected, countries with a high proportion of women having a child before 18 years of age also have the highest rates of adolescent fertility. However, a more consistent reduction in AFR, when not accompanied by the same reduction in early childbearing, suggests that the average number of children born to adolescent girls (represented by the fertility rates) decreased in most countries, but not the occurrence of the first birth before 18 years of age. A notable case was Colombia, where we observed an increase in the proportion of women experiencing early childbearing across generations, while AFR decreased.

We also explored inequalities between population subgroups. Adolescent girls from families in the lowest wealth quintiles, living in the rural areas, and from indigenous and Afro-descendant groups were disproportionately affected by adolescent motherhood. The higher proportion of adolescent births to poorer mothers is in line with other work from the region that showed broader socioeconomic inequalities persist in rates of adolescent pregnancies.^10^ Adolescent childbearing in the most disadvantaged groups further increases their vulnerabilities over the life course, reinforcing the socioeconomic inequalities.^24^

The LAC region is one of the most urbanised in the world, with large social and income inequalities.^25^ The scarcity of published evidence covering ethnic inequalities in adolescent childbearing reinforces the statistical invisibility of ethnic groups in the region. This is relevant, given that ethnicity-based inequalities and poverty are closely linked, and cultural traits and ethnic background might play a part in the value of early motherhood and reproductive autonomy.^22^, ^26^ Women from ethnic minorities tend to experience poor social and economic conditions, which could contribute to greater inequity in access to family planning services and contraceptive methods.^22^, ^23^, ^27^

Our forecasting exercise for AFR suggested that by 2030 most of the study countries would have rates between 45 and 89 births per 1000 women-years if the current trends persist. The Strategic Plan of the Pan American Health Organization, 2020–25, has established a target of reducing the AFR by 10% between 2015 and 2025, equivalent to an annual AAC of –0·7 per 1000.^28^ Based on our presented results, all nine study countries could achieve this goal. However, in seven countries the most recent AFR estimate for the wealthiest 60% group is below 60 livebirths per 1000 adolescents, and we observed that by 2030, the end of the SDG era, only three countries in the sample will present an AFR at the national level that will be lower than this figure, as the predicted national estimates for 2030 range from 45–89 livebirths per 1000 women-years. Moreover, given that in all countries the observed reductions in AFR are below three births per 1000 women per year, it would take more than 10 years to reduce the AFR by another 30 births per 1000 women-years. Results for Ecuador need to be taken with caution as only two surveys are available with a 6-year interval, providing estimates that might be unstable.

The LAC region is culturally diverse and inequitable, and the factors associated with adolescent childbearing vary widely between and within countries.^29^, ^30^ Regional and country-level efforts must ensure that investments reach the most vulnerable adolescents first through the application of equity-based and culturally appropriate approaches.^31^

Gender-based and socioeconomic inequalities have been highlighted as key drivers of adolescent motherhood by increasing child marriage, preventing girls’ access to schooling and attendence, restricting their career possibilities, and limiting health care and information on sexual and reproductive health. 1, 32

Another important driver of early childbearing is the lack of access to modern family planning methods. A modelling approach based on DHS data between 1986 and 2015 suggests that increasing contraceptive use by adolescents has helped reduce adolescent fertility by 6·8% in the region.^33^ It has been estimated that less than 30% of girls aged 15–19 years in LAC were using a modern contraceptive method in 2019.^24^, ^34^ Also, inequalities in the use of contraceptives in the region have been reported as the highest among the world regions.^35^ This could be due to social norms that limit the use of contraception by adolescents, or because of the lack of suitable and adequate services, information, and methods for adolescents to prevent an undesired pregnancy.^31^

WHO has published a set of recommendations to prevent early pregnancy, which cover numerous possible actions to be taken, including preventing early marriage (before 18 years of age), increasing knowledge and understanding of the importance of pregnancy prevention, increasing sexual education, contraceptive counselling and use, and preventing coerced sex.^36^ Broader actions aimed at investment in women's and girls’ empowerment and improvement efforts to retain girls in school and to strengthen career paths or job opportunities for young women are also recommended. Additionally, the associated poor reproductive outcomes could be prevented by reducing unsafe abortions by providing adequate care, offering and promoting postpartum and post-abortion contraception, and increasing the use of skilled antenatal, childbirth, and postnatal care.^36^ National programmes providing evidence in reducing adolescent childbearing and subnational inequalities need to scale up, ensuring that girls living in conditions of vulnerability are reached effectively.^37^ Efforts must also engage men and boys to become partners in the process of avoiding unintended pregnancies, by also offering them adequate sexual and reproductive health education, and improving their understanding of gender-related issues that influence women's and girls’ lives via tailored campaigns.^37^ This needs to be addressed through intersectoral national and regional collaboration. It is noteworthy that lowering the occurrence of adolescent childbearing is central to achieving multiple SDGs, as it will impact other development indicators, for instance, by reducing maternal and infant mortality.

Country-specific initiatives to reduce adolescent childbearing have been implemented.^30^ One review of public policies in the LAC region has suggested that policies related to conditional cash transfers and compulsory education have the strongest evidence of correlation with adolescent pregnancy prevention.^38^ Some examples include subsidies for girls attending and completing their education; training focused on improvements in self-esteem, self-efficacy, and enhancement of life plans for adolescents; and national and intersectoral action plans for preventing adolescent childbearing.^39^ The success of these programmes underscores that ensuring adolescent girls stay in school, providing them with life-skills training, and generating awareness about the impact of unintended early childbearing can be effective.^39^ As a next step, expanding the initiatives to men and boys can help to improve the circumstance in which all adolescents live. In addition to providing sexual education in schools and health facilities, it is crucial to ensure access to these programmes for all adolescents regardless of gender. This should be done in conjunction with addressing the social determinants of the observed inequalities, working to reduce overall socioeconomic disparities, and breaking the intergenerational cycles of poverty.

This analysis has some limitations. Both indicators include livebirths only, excluding the occurrence of pregnancies that end in miscarriage or abortion. In low-income and middle-income countries, estimates suggest that up to half of adolescent girls’ unintended pregnancies end in abortions. In 2019, the number of unsafe abortions in girls aged 15–19 years in LAC countries was around 876 000. Indicators covering adolescent pregnancy, regardless of the outcome, can provide a more comprehensive picture of the issue in the region.^30^ Although differences by age at the first birth among adolescents might lead to different outcomes, we chose not to disaggregate the estimates further to maintain the groups with a reasonable size for the equity analyses.^10^ Also, the data are cross-sectional, and women and household characteristics were recorded at the time of the survey, not at the time of birth. Due to the unavailability of information on ethnicity for several surveys, especially those conducted earliest, we would not be able to include all countries in this set of analyses. Furthermore, even for countries that have information for two or more surveys, we would not be comparing the same survey-years for all stratifiers, since this information is missing for some of them. For Ecuador, the availability of only two surveys close to each other in time might have led to an overestimation of the decline of adolescent childbearing over time. Finally, we used a linear model to estimate an AAC in the indicators of interest. Despite our interest being solely on a single average estimate of change, the speed of change might not have been constant, and this was not considered.

Adolescence is the phase of life between childhood and adulthood, from 10 to 19 years of age, and studies on reproductive health tend to include only those women commonly defined as being of reproductive age, between 15 and 49 years. Therefore, the operational definition of adolescent fertility rates, as adopted by several scholars and organisations, is necessarily the number of births of women aged between 15 and 19 years. More recently, estimates of fertility rates for girls aged between 10 and 14 years have been incorporated in health and development monitoring initiatives, including the SDGs (Indicator 3.7.2). It has been estimated that most LAC countries present, on average, 1–5 births per 1000 girls aged from 10 to 14 years per year.^40^ Although we recognise the greater vulnerability of younger girls, to keep the indicators consistent, we used the 15–19-year age group because it has been benchmarked for almost all countries for monitoring purposes. Regarding early childbearing, we chose the operational definition as used by international organisations, which does not include adolescent girls aged 19 years. Also, albeit including different age groups in their definitions and not being directly comparable, the indicators provide a useful landscape of regional trends and complementary information on reproductive health among adolescent girls.

Key strengths of this study are the inclusion of 21 of 33 countries in the LAC region for analysis of early childbearing, which has allowed us to update previous studies by including a much larger number of geographies, and expanding the approach for analysis of socioeconomic inequalities. We were also able to harmonise DHS, Multiple Indicator Cluster Surveys, and other country-specific surveys to include as much as possible of this elevated number of countries from the LAC region. The possibility of including both early childbearing and adolescent fertility rate indicators is also a positive aspect of the study, covering two different measures of progress and development concerning adolescent sexual and reproductive health in general, and motherhood in particular. Finally, disaggregated data that examine patterns and trends for different groups are needed to enable programmes to be focused on those most at risk.

In conclusion, our results indicate a reduction in the average number of births among adolescents, represented by the fertility rates for adolescent girls, along with a decrease in the proportion of young women who become adolescent mothers in some countries, but not in all. Reducing adolescent childbearing and addressing its multiple underlying factors are essential for improving sexual and reproductive health and the social and economic wellbeing of this group. This will require giving girls a better perspective in life, focusing on encouraging schooling, delaying childbearing, ensuring access to family planning, strengthening reproductive health rights (including access to safe abortion and contraceptives), and providing general high-quality accessible health services. National policies need to guarantee the provision of sexual education to both boys and girls, increase social and economic support, and deliver quality health services. These are necessary and urgent actions to be taken by policymakers and should be in conjunction with broader social changes, including the recognition of the rights, capacities, and needs of adolescents, particularly those from disadvantaged groups.

## Data sharing

All data are publicly available and can be downloaded from the websites of the programmes conducting the surveys.

## Declaration of interests

We declare no competing interests.

## Supplementary Materials

Spanish translation of the abstract

Portuguese translation of the abstract
