# Supplementary material for: Trends and inequities in adolescent childbearing in Latin American and Caribbean countries across generations and over time: a population-based study
Source: Lancet Child Adolesc Health. 2023 Jun;7(6):392–404. doi: 10.1016/S2352-4642(23)00077-9 (PMC10191863; doi:10.1016/S2352-4642(23)00077-9)
Supplement: Spanish translation of the abstract [file mmc1.pdf]

THE LANCET
Child & Adolescent Health

Supplementary appendix 1

This translation in Spanish was submitted by the authors and we reproduce it as supplied. It has not been peer reviewed. *The Lancet*'s editorial processes have only been applied to the original in English, which should serve as reference for this manuscript.

Los autores nos proporcionaron esta traducción al español y la reproducimos tal como nos fue entregada. No la hemos revisado. Los procesos editoriales de *The Lancet* se han aplicado únicamente al original en inglés, que debe servir de referencia para este manuscrito.

Supplement to: Sanhueza A, Costa JC, Mújica OJ, et al. Trends and inequities in adolescent childbearing in Latin American and Caribbean countries across generations and over time: a population-based study. *Lancet Child Adolesc Health* 2023; **7:** 392–404.

Tendencias e inequidades en la maternidad adolescente en países de América Latina y el Caribe a través de generaciones y a lo largo del tiempo: un estudio de base poblacional

Resumen

Antecedentes América Latina y el Caribe exhibe la segunda tasa de fecundidad adolescente más alta del mundo, sólo después de África subsahariana, y ha alcanzado la tercera posición a nivel mundial en la incidencia de maternidad en la adolescencia. Nuestro objetivo fue explorar las tendencias e inequidades en la maternidad adolescente en la región.

Métodos Utilizamos encuestas de hogares representativas a nivel nacional de países de América Latina y el Caribe para abordar las tendencias en la maternidad temprana (proporción de mujeres que tienen su primer hijo antes de los 18 años) a través de generaciones y en las tasas de fecundidad adolescente (TFA; nacimientos por 1000 mujeres de 15 a 19 años) a lo largo del tiempo. Para la maternidad temprana, analizamos la encuesta más reciente realizada desde 2010 en 21 países (2010-20); para la TFA, analizamos nueve países con dos o más encuestas, la más reciente a partir de 2010. Para ambos indicadores, se utilizó la regresión de mínimos cuadrados ponderados por varianza para estimar los cambios absolutos promedio (CAP) a nivel nacional y por riqueza (40% más bajo vs 60% más alto), residencia urbana versus rural y etnicidad.

Hallazgos Entre los 21 países estudiados, observamos una disminución en la maternidad temprana a través de generaciones en 13 de ellos, con disminuciones en el CAP que varían de -0,6 puntos porcentuales (IC del 95%: -1,1 a -0,1) en Haití a -2,7 puntos porcentuales (-4,0 a -1,4) en Santa Lucía. Observamos un aumento a través de generaciones en Colombia (1,2 puntos porcentuales [0,8 a 1,5]) y México (1,3 puntos porcentuales [0,5 a 2,0]) y ningún cambio en Bolivia y Honduras. La disminución más rápida de la maternidad temprana se produjo entre las mujeres rurales, mientras que no se observó un patrón claro para los grupos de riqueza. Se encontraron estimaciones decrecientes de las generaciones más antiguas a las más jóvenes entre los afrodescendientes y lo grupo no afrodescendientes/no indígenas, pero los resultados fueron mixtos para las personas indígenas. Los nueve países con datos para TFA presentaron reducciones a lo largo del tiempo (-0,7 a -6,5 nacimientos por 1000 mujeres por año), siendo las disminuciones más pronunciadas las observadas en Ecuador, Guyana, Guatemala y República Dominicana. En general, las adolescentes en áreas rurales y las adolescentes más pobres tuvieron las mayores reducciones en la TFA. Si las tendencias actuales persisten, para 2030 la mayoría de los países tendrán valores de TFA entre 45 y 89 nacimientos por 1000 mujeres, con notables desigualdades asociadas a riqueza.

Interpretación Nuestros resultados indican una reducción en la TFA en países de América Latina y el Caribe que no fue necesariamente acompañada por una disminución en la maternidad temprana en general. Se observaron grandes desigualdades tanto entre países como al interior de los países, sin una reducción clara a lo largo del tiempo. Comprender las tendencias en la maternidad adolescente y sus determinantes es esencial para diseñar y planificar programas que garanticen las reducciones deseadas en las tasas y las brechas entre los subgrupos de población.

Financiamiento OPS, Fundación Bill & Melinda Gates, y Wellcome Trust.
