# Supplementary material for: Trends and inequities in adolescent childbearing in Latin American and Caribbean countries across generations and over time: a population-based study
Source: Lancet Child Adolesc Health. 2023 Jun;7(6):392–404. doi: 10.1016/S2352-4642(23)00077-9 (PMC10191863; doi:10.1016/S2352-4642(23)00077-9)
Supplement: Portuguese translation of the abstract [file mmc2.pdf]

THE LANCET
Child & Adolescent Health

Supplementary appendix 2

This translation in Portuguese was submitted by the authors and we reproduce it as supplied. It has not been peer reviewed. *The Lancet*'s editorial processes have only been applied to the original in English, which should serve as reference for this manuscript.

Esta tradução em português foi submetida pelos autores e nós não fizemos quaisquer alterações. Esta versão não foi revista por pares. O processo editorial do The Lancet só foi aplicado à versão original em inglês, que deve servir como referência para este artigo.

Supplement to: Sanhueza A, Costa JC, Mújica OJ, et al. Trends and inequities in adolescent childbearing in Latin American and Caribbean countries across generations and over time: a population-based study. *Lancet Child Adolesc Health* 2023; **7:** 392–404.

Tendências e iniquidades na maternidade na adolescência em países da América Latina e do Caribe ao longo de gerações e do tempo: um estudo de base populacional

Resumo

Antecedentes A América Latina e o Caribe apresentam a segunda maior taxa de fecundidade na adolescência do mundo, atrás apenas da África Subsaariana, e alcançou a terceira posição global na incidência de maternidade na adolescência. Nosso objetivo foi explorar tendências e desigualdades na maternidade adolescente na região.

Métodos Utilizamos inquéritos domiciliares nacionalmente representativos de países da América Latina e do Caribe para analisar as tendências da maternidade precoce (proporção de mulheres que tiveram o primeiro filho nascido vivo antes dos 18 anos) ao longo das gerações e das taxas de fecundidade na adolescência (TFA; nascimentos por 1000 mulheres com idade entre 15 e 19 anos) ao longo do tempo. Para maternidade precoce, analisamos o inquérito mais recente realizado desde 2010 em 21 países (2010-20); para TFA, analisamos nove países com dois ou mais inquéritos, sendo o mais recente realizado a partir de 2010. Para ambos os indicadores, foi utilizada a regressão por quadrados mínimos ponderada pela variância para estimar as mudanças absolutas médias em nível nacional e por riqueza (40% mais baixos vs 60% mais altos), residência urbana vs rural, e etnia.

Resultados Entre os 21 países estudados, observamos uma diminuição da maternidade precoce ao longo das gerações em 13 deles, com declínios que variaram entre -0,6 pontos percentuais (IC 95% -1,1 a -0,1) no Haiti e -2,7 pontos percentuais (-4,0 a -1,4) em Santa Lúcia. Observamos aumentos ao longo das gerações na Colômbia (1-2 pontos percentuais [0,8 a 1,5]) e no México (1,3 pontos percentuais [0,5 a 2,0]) e nenhuma alteração na Bolívia e Honduras. O declínio mais rápido da maternidade precoce ocorreu entre as mulheres rurais, enquanto não se observou um padrão claro para os grupos de riqueza. Foram encontradas estimativas decrescentes das gerações mais velhas para as mais novas entre os grupos afrodescendentes e não afrodescendentes/não indígenas, mas os resultados foram mistos para os povos indígenas. Todos os nove países com dados para a TFA apresentaram reduções ao longo do tempo (-0,7 a -6,5 nascimentos por 1000 mulheres por ano), com os declínios mais acentuados observados no Equador, Guiana, Guatemala e República Dominicana. Em geral, adolescentes das zonas rurais e adolescentes mais pobres registaram as maiores reduções na taxa de fecundidade. Se as tendências atuais persistirem, em 2030 a maioria dos países apresentará valores de TFA entre 45 e 89 nascimentos por 1000 mulheres, com notáveis desigualdades relacionadas à riqueza.

Interpretação Nossos resultados indicam uma redução da TFA nos países da América Latina e do Caribe, que não foi necessariamente acompanhada por uma diminuição da maternidade precoce em geral. Foram observadas grandes desigualdades entre países e dentro de cada país, sem uma redução clara ao longo do tempo. Compreender as tendências da maternidade na adolescência e os seus determinantes é essencial para desenhar e planejar programas que garantam as reduções desejadas nas taxas e nas desigualdades nos subgrupos da população.

Financiamento OPAS, Bill & Melinda Gates Foundation, e Wellcome Trust.
