# Supplementary material for: Trends and inequities in adolescent childbearing in Latin American and Caribbean countries across generations and over time: a population-based study
Source: Lancet Child Adolesc Health. 2023 Jun;7(6):392–404. doi: 10.1016/S2352-4642(23)00077-9 (PMC10191863; doi:10.1016/S2352-4642(23)00077-9)
Supplement: Supplementary appendix 3 [file mmc3.pdf]

THE LANCET
Child & Adolescent Health

Supplementary appendix 3

This appendix formed part of the original submission and has been peer reviewed. We post it as supplied by the authors.

Supplement to: Sanhueza A, Costa JC, Mújica OJ, et al. Trends and inequities in adolescent childbearing in Latin American and Caribbean countries across generations and over time: a population-based study. *Lancet Child Adolesc Health* 2023; **7:** 392–404.

Supplementary Materials – Trends and inequities in adolescent childbearing in Latin American and Caribbean countries across generations and over time: a population-based study

Supplementary Table S1 – Search strategy ... 2

Supplementary Table S2 – List of surveys (year and type) included in the analyses. ... 3

Supplementary Table S3 – Early childbearing (percentage of women who had their first birth before 18 years) by generation, unweighted sample size, and corresponding average absolute change (AAC) across groups at the national level in Latin American and Caribbean countries. ... 5

Supplementary Table S4 – Early childbearing (percentage of women who had their first birth before 18 years) by generation, unweighted sample size, and corresponding average absolute change across groups, by wealth (bottom 40% and top 60%) in Latin American and Caribbean countries. ... 6

Supplementary Table S5 – Early childbearing (percentage of women who had their first birth before 18 years) by generation, unweighted sample size, and corresponding average absolute change across groups by urban/rural residence, in Latin American and Caribbean countries. ... 8

Supplementary Table S6 – Early childbearing (percentage of women who had their first birth before 18 years) by generation, unweighted sample sizes, and corresponding average absolute change across groups, by ethnicity, in Latin American and Caribbean countries. ... 10

Supplementary Table S7 – Adolescent fertility rate (births per 1,000 women aged 15-19 years) by wealth (bottom 40% and top 60%) and p value for interaction between wealth and survey year based on country-specific variance weighted linear regression models. ... 12

Supplementary Table S8 – Annual average absolute change (AAC) in adolescent fertility rate (births per 1,000 women aged 15-19 years in the five years preceding the survey) over time at the national level and by wealth groups (bottom 40% and top 60%) and predicted values for 2030. ... 14

Supplementary Figure S1 – Early childbearing (percentage of women aged 20-24 years who had their first birth before 18 years) and adolescent fertility rates (births per 1,000 women aged 15-19 years in the five years preceding the survey) with a regression line (Pearson's correlation = 0·83, p = 0.001). ... 15

Supplementary Figure S2 – Average absolute change in early childbearing (women having a first birth before the age of 18) in percentage points across generations at the national level in Latin American and Caribbean countries. ... 16

Supplementary Figure S3 – Trends in adolescent fertility rate (births per 1,000 women aged 15-19 years in the five years preceding the survey) at the national level and corresponding annual average absolute change in Latin American and Caribbean countries. ... 17

Supplementary Figure S4 – Trends in adolescent fertility rate (births per 1,000 women aged 15-19 years in the five years preceding the survey) by wealth groups and corresponding annual average absolute change in Latin American and Caribbean countries. ... 18

Supplementary Figure S5 – Trends in adolescent fertility rate (births per 1,000 women aged 15-19 years in the five years preceding the survey) by urban/rural residence and corresponding annual average absolute change in Latin American and Caribbean countries. ... 19

Supplementary Figure S6 – Trends in adolescent fertility rate (births per 1,000 women aged 15-19 years in the five years preceding the survey) by ethnicity (Indigenous, Afrodescendant, Other non-Indigenous/non-Afrodescendant groups) in Latin American and Caribbean countries. ... 20

Supplementary Table S1 – Search strategy

#1	(ADOLESCENT OR YOUNG ADULT) AND (PREGNANCY OR REPRODUCTIVE HEALTH OR BIRTH RATE)<br><br>AND<br><br>("LATIN AMERICA" OR CARIBBEAN OR "ANTIGUA AND BARBUDA" OR ARGENTINA OR ARUBA OR BAHAMAS OR BARBADOS OR BELIZE OR BOLIVIA OR BRAZIL OR "BRITISH VIRGIN ISLANDS" OR "CAYMAN ISLANDS" OR CAYMAN OR CHILE OR COLOMBIA OR "COSTA RICA" OR CUBA OR CURACAO OR DOMINICA OR "DOMINICAN REPUBLIC" AND ECUADOR OR "EL SALVADOR" OR GRENADA OR GUATEMALA OR GUYANA OR HAITI OR HONDURAS OR JAMAICA OR MEXICO OR NICARAGUA OR PANAMA OR PARAGUAY OR PERU OR "PUERTO RICO" AND "SINT MAARTEN" AND "ST. KITTS AND NEVIS" OR "ST. LUCIA" OR "ST. MARTIN" OR "ST. VINCENT AND THE GRENADINES" OR SURINAME OR "TRINIDAD AND TOBAGO" OR "TURKS AND CAICOS ISLANDS" OR "TURKS AND CAICOS" OR URUGUAY OR VENEZUELA OR "VIRGIN ISLANDS")
#2	ADOLESCENT PREGNANCY<br><br>AND<br><br>("LATIN AMERICA" OR CARIBBEAN OR "ANTIGUA AND BARBUDA" OR ARGENTINA OR ARUBA OR BAHAMAS OR BARBADOS OR BELIZE OR BOLIVIA OR BRAZIL OR "BRITISH VIRGIN ISLANDS" OR "CAYMAN ISLANDS" OR CAYMAN OR CHILE OR COLOMBIA OR "COSTA RICA" OR CUBA OR CURACAO OR DOMINICA OR "DOMINICAN REPUBLIC" AND ECUADOR OR "EL SALVADOR" OR GRENADA OR GUATEMALA OR GUYANA OR HAITI OR HONDURAS OR JAMAICA OR MEXICO OR NICARAGUA OR PANAMA OR PARAGUAY OR PERU OR "PUERTO RICO" AND "SINT MAARTEN" AND "ST. KITTS AND NEVIS" OR "ST. LUCIA" OR "ST. MARTIN" OR "ST. VINCENT AND THE GRENADINES" OR SURINAME OR "TRINIDAD AND TOBAGO" OR "TURKS AND CAICOS ISLANDS" OR "TURKS AND CAICOS" OR URUGUAY OR VENEZUELA OR "VIRGIN ISLANDS")

Supplementary Table S2 – List of surveys (year and type) included in the analyses.

Country	Survey	
	Year	Type
Argentina	2019	MICS
Barbados	2012	MICS
Belize	2015	MICS
Bolivia	1994	DHS
	1998	DHS
	2003	DHS
	2008	DHS
	2016	EDSA
Colombia	1995	DHS
	2000	DHS
	2005	DHS
	2010	DHS
	2015	DHS
Costa Rica	2018	MICS
Cuba	2019	MICS
Dominican Republic	1996	DHS
	1999	DHS
	2002	DHS
	2007	DHS
	2013	DHS
	2014	MICS
	2019	MICS
Ecuador	2012	ENSANUT
	2018	ENSANUT
El Salvador	2014	MICS
Guatemala	1995	DHS
	1998	DHS
	2014	DHS
Guyana	2009	DHS
	2014	MICS
	2019	MICS
Haiti	1994	DHS
	2000	DHS
	2005	DHS
	2012	DHS
	2016	DHS
Honduras	2005	DHS
	2011	DHS
	2019	MICS
Jamaica	2011	MICS
Mexico	2015	MICS
Paraguay	2016	MICS
Peru	1996	DHS
	2000	DHS
	2004	DHS
	2005	DHS
	2006	DHS
	2007	DHS
	2008	DHS
	2009	DHS
	2010	DHS
	2011	DHS

Country	Survey	
	Year	Type
	2012	DHS
	2013	ENDES/DHS
	2014	ENDES/DHS
	2015	ENDES/DHS
	2016	ENDES/DHS
	2017	ENDES/DHS
	2018	ENDES/DHS
	2019	ENDES/DHS
Saint Lucia	2012	MICS
Suriname	2018	MICS
Trinidad and Tobago	2011	MICS

Supplementary Table S3 – Early childbearing (percentage of women who had their first birth before 18 years) by generation, unweighted sample size, and corresponding average absolute change (AAC) across groups at the national level in Latin American and Caribbean countries.

Survey	Women who had their first birth before 18 years by age groups												AAC (95%CI)
	45-49 years		40-44 years		35-39 years		30-34 years		25-29 years		20-24 years		
	% (95%CI)	N	% (95%CI)	N	% (95%CI)	N	% (95%CI)	N	% (95%CI)	N	% (95%CI)	N	
Argentina (2019)	15.3 (12.5; 18.7)	1,161	13.6 (11.0; 16.8)	1516	16.5 (13.7; 19.7)	1802	12.7 (10.5; 15.1)	1944	15.0 (12.5; 17.8)	2123	14.1 (11.9; 16.6)	1997	-0.2 (-0.8; 0.5)
Barbados (2012)	14.4 (9.9; 20.6)	244	11.1 (7.1; 17.0)	208	13.0 (9.0; 18.3)	230	7.3 (4.4; 11.9)	244	8.9 (5.8; 13.5)	229	6.7 (3.9; 11.5)	192	-1.4 (-2.5; -0.4)
Belize (2015)	23.7 (18.7; 29.6)	389	24.1 (19.4; 29.5)	432	24.3 (19.7; 29.7)	550	21.0 (17.6; 24.8)	740	19.7 (16.4; 23.5)	817	17.3 (14.5; 20.5)	872	-1.5 (-2.5; -0.5)
Bolivia (2016)	18.6 (15.4; 22.4)	1,117	15.5 (13.2; 18.3)	1345	15.6 (13.5; 18.0)	1594	17.9 (15.9; 20.2)	1686	15.8 (13.8; 18.0)	1795	17.1 (14.9; 19.5)	1866	0.0 (-0.6; 0.6)
Colombia (2015)	14.1 (12.7; 15.6)	4,315	16.6 (15.0; 18.2)	4273	19.4 (17.5; 21.5)	4655	21.8 (20.0; 23.8)	5076	20.0 (18.2; 22.0)	5260	19.5 (17.9; 21.1)	5796	1.2 (0.8; 1.5)
Costa Rica (2018)	13.6 (11.0; 16.8)	726	20.6 (16.9; 25.0)	822	20.3 (17.0; 24.0)	1074	15.6 (12.6; 19.3)	1294	13.9 (11.5; 16.7)	1355	13.1 (10.8; 15.7)	1235	-0.6 (-1.3; 0.0)
Cuba (2019)	16.8 (13.7; 20.5)	1,199	15.4 (11.4; 20.4)	975	11.3 (8.2; 15.4)	1095	13.3 (10.4; 16.7)	1692	7.9 (6.0; 10.3)	1773	10.3 (8.0; 13.2)	1309	-1.5 (-2.3; -0.8)
Dominican Republic (2019)	20.8 (18.7; 23.1)	2,520	22.8 (20.8; 24.9)	2684	27.6 (25.4; 29.9)	2907	24.9 (22.6; 27.5)	3038	20.4 (18.6; 22.3)	3618	20.4 (18.7; 22.3)	3701	-0.5 (-0.9; 0.0)
Ecuador (2018)	14.6 (12.9; 16.5)	3,535	18.4 (16.3; 20.7)	4407	21.0 (19.0; 23.0)	5613	21.8 (20.1; 23.7)	6289	22.1 (20.2; 24.0)	6717	10.9 (9.7; 12.2)	7062	-0.9 (-1.2; -0.5)
El Salvador (2014)	23.9 (21.1; 27.0)	1,149	23.0 (20.3; 26.0)	1310	24.1 (21.4; 26.9)	1676	25.3 (22.7; 28.2)	1918	19.1 (17.0; 21.3)	2195	18.2 (16.4; 20.1)	2622	-1.3 (-1.9; -0.8)
Guatemala (2014)	24.0 (21.9; 26.4)	2,146	23.0 (21.1; 25.0)	2516	23.8 (22.2; 25.6)	3077	24.3 (22.8; 25.9)	3581	21.0 (19.5; 22.6)	3997	20.2 (18.7; 21.7)	4826	-0.8 (-1.2; -0.4)
Guyana (2019)	23.1 (19.2; 27.6)	655	20.5 (16.2; 25.7)	688	21.8 (17.8; 26.4)	696	21.9 (17.8; 26.5)	725	14.8 (11.7; 18.6)	1012	13.5 (10.3; 17.4)	1103	-2.0 (-2.9; -1.1)
Haiti (2016)	15.6 (13.2; 18.3)	1,217	17.2 (15.0; 19.7)	1308	15.8 (13.8; 18.1)	1645	16.2 (14.4; 18.2)	1919	13.9 (12.0; 16.0)	2202	13.6 (12.1; 15.3)	2773	-0.6 (-1.1; -0.1)
Honduras (2019)	21.4 (19.3; 23.7)	1,854	25.7 (23.6; 27.9)	2255	27.4 (25.3; 29.6)	2386	25.8 (23.9; 27.9)	2625	24.0 (22.2; 26.0)	3041	25.8 (24.0; 27.6)	3365	0.4 (-0.1; 0.9)
Jamaica (2011)	27.6 (23.3; 32.4)	595	25.6 (21.6; 30.1)	659	21.9 (18.8; 25.4)	706	23.7 (19.7; 28.2)	682	19.5 (15.9; 23.7)	722	14.9 (12.2; 18.0)	753	-2.4 (-3.3; -1.6)
Mexico (2015)	13.0 (10.0; 16.6)	1,114	17.2 (14.0; 20.8)	1320	13.9 (11.7; 16.5)	1561	18.7 (15.2; 22.9)	1873	16.6 (13.5; 20.2)	2171	20.5 (17.8; 23.5)	2280	1.3 (0.5; 2.0)
Paraguay (2016)	14.0 (10.8; 17.8)	579	16.8 (13.4; 20.8)	654	19.6 (16.2; 23.5)	894	16.8 (13.8; 20.2)	1250	15.3 (12.7; 18.2)	1293	14.8 (12.5; 17.4)	1342	-0.2 (-0.9; 0.5)
Peru (2020)	13.4 (11.7; 15.3)	3,060	13.9 (12.2; 15.7)	3982	14.9 (13.5; 16.5)	4869	14.3 (13.0; 15.7)	5153	15.0 (13.6; 16.5)	5177	11.4 (10.2; 12.7)	4665	-0.4 (-0.7; -0.0)
Saint Lucia (2012)	23.5 (17.1; 31.5)	172	20.6 (15.0; 27.5)	183	15.0 (9.5; 22.8)	158	18.1 (12.3; 25.9)	160	12.1 (7.2; 19.6)	178	9.3 (6.0; 14.0)	189	-2.7 (-4.0; -1.4)
Suriname (2018)	18.8 (15.4; 22.6)	732	15.0 (11.9; 18.7)	756	15.7 (12.1; 20.3)	970	16.5 (13.8; 19.6)	1147	13.7 (11.1; 16.8)	1138	13.2 (11.0; 15.8)	1042	-0.9 (-1.6; -0.2)
Trinidad and Tobago (2011)	11.9 (9.4; 15.0)	606	10.1 (7.7; 13.2)	484	11.2 (8.6; 14.4)	549	7.7 (5.6; 10.4)	596	7.1 (5.2; 9.6)	697	6.3 (4.5; 8.8)	632	-1.1 (-1.7; -0.6)

AAC = average absolute change in percentage points; negative values indicate a reduction in the percentage of women who had a first birth before 18 years over generations whereas positive numbers indicate an increase in the figures.

Supplementary Table S4 – Early childbearing (percentage of women who had their first birth before 18 years) by generation, unweighted sample size, and corresponding average absolute change across groups, by wealth (bottom 40% and top 60%) in Latin American and Caribbean countries.

Survey	Level	Women who had their first birth before 18 years by age groups												AAC (95%CI)
		45-49 years		40-44 years		35-39 years		30-34 years		25-29 years		20-24 years		
		% (95%CI)	N	% (95%CI)	N	% (95%CI)	N	% (95%CI)	N	% (95%CI)	N	% (95%CI)	N	
Argentina (2019)	Bottom 40%	29.0 (22.5; 36.5)	420	20.0 (15.4; 25.4)	575	23.6 (18.9; 29.1)	680	16.3 (13.0; 20.2)	832	21.6 (17.7; 26.2)	1010	20.5 (17.0; 24.6)	1004	-0.7 (-1.9; 0.4)
	Top 60%	9.1 (6.9; 12.0)	741	9.6 (6.9; 13.4)	941	12.0 (8.9; 16.0)	1122	9.9 (7.3; 13.4)	1112	7.9 (5.8; 10.7)	1113	8.1 (5.8; 11.2)	993	-0.3 (-1.0; 0.3)
Barbados (2012)	Bottom 40%	21.7 (13.7; 32.8)	81	16.4 (9.4; 27.0)	68	27.4 (16.5; 42.0)	67	7.4 (3.4; 15.5)	81	14.4 (8.2; 24.0)	86	6.6 (2.5; 16.3)	64	-2.8 (-4.6; -0.9)
	Top 60%	11.0 (6.5; 17.9)	163	8.4 (4.3; 16.0)	140	6.8 (3.8; 12.0)	163	7.3 (3.8; 13.6)	163	6.0 (3.0; 11.8)	143	6.8 (3.5; 12.9)	128	-0.7 (-1.9; 0.4)
Belize (2015)	Bottom 40%	31.3 (22.7; 41.4)	149	29.6 (21.3; 39.6)	151	30.4 (22.4; 39.9)	203	32.7 (26.1; 40.1)	261	29.4 (23.1; 36.4)	310	24.9 (20.0; 30.5)	370	-1.2 (-3.0; 0.5)
	Top 60%	19.9 (14.3; 27.1)	240	21.8 (16.2; 28.6)	281	20.9 (16.0; 26.9)	347	15.1 (11.6; 19.4)	479	13.7 (10.4; 17.7)	507	12.5 (9.5; 16.2)	502	-2.0 (-3.1; -0.9)
Bolivia (2016)	Bottom 40%	19.5 (13.9; 26.6)	549	18.6 (14.7; 23.3)	550	22.1 (18.3; 26.5)	666	25.0 (21.3; 29.1)	707	24.6 (21.0; 28.7)	730	25.6 (21.3; 30.5)	746	1.6 (0.4; 2.7)
	Top 60%	18.0 (14.4; 22.3)	568	14.1 (11.3; 17.6)	795	12.3 (10.0; 15.1)	928	14.1 (11.8; 16.9)	979	11.9 (9.6; 14.5)	1065	13.0 (10.8; 15.7)	1120	-0.6 (-1.3; 0.1)
Colombia (2015)	Bottom 40%	22.9 (20.4; 25.7)	2189	24.9 (22.4; 27.5)	2323	29.3 (26.7; 32.0)	2507	30.7 (28.3; 33.2)	2736	29.1 (26.8; 31.4)	2902	30.2 (27.7; 32.8)	3225	1.4 (0.8; 2.0)
	Top 60%	9.9 (8.5; 11.6)	2126	12.1 (10.3; 14.2)	1950	14.1 (12.0; 16.6)	2148	17.2 (14.9; 19.9)	2340	14.3 (11.8; 17.1)	2358	11.8 (10.0; 13.7)	2571	0.5 (0.1; 1.0)
Costa Rica (2018)	Bottom 40%	21.0 (15.5; 27.8)	319	29.4 (22.9; 36.9)	399	30.8 (25.2; 36.9)	486	26.9 (21.2; 33.4)	602	22.4 (18.5; 26.9)	691	23.4 (19.1; 28.3)	677	-0.5 (-1.8; 0.8)
	Top 60%	10.3 (7.4; 14.1)	407	14.6 (10.7; 19.8)	423	14.8 (11.0; 19.6)	588	8.7 (6.3; 11.8)	692	9.0 (6.2; 13.0)	664	6.5 (4.1; 10.1)	558	-1.1 (-1.9; -0.3)
Cuba (2019)	Bottom 40%	27.5 (21.3; 34.8)	470	24.4 (18.1; 31.9)	373	16.5 (10.4; 25.1)	364	22.2 (16.7; 28.8)	551	11.8 (8.6; 16.0)	665	17.8 (13.2; 23.6)	490	-2.6 (-4.0; -1.2)
	Top 60%	9.3 (6.9; 12.5)	729	9.4 (5.9; 14.6)	602	7.9 (5.1; 12.0)	731	8.3 (5.8; 11.7)	1141	5.4 (3.4; 8.3)	1108	6.8 (4.5; 10.1)	819	-0.7 (-1.4; -0.0)
Dominican Republic (2019)	Bottom 40%	27.6 (24.0; 31.6)	1057	29.2 (26.0; 32.6)	1107	37.0 (33.0; 41.2)	1268	37.4 (33.5; 41.4)	1330	31.8 (28.3; 35.4)	1617	29.4 (26.4; 32.5)	1780	0.3 (-0.5; 1.1)
	Top 60%	17.2 (14.7; 20.1)	1463	19.7 (17.3; 22.4)	1577	22.7 (20.2; 25.5)	1639	18.3 (15.7; 21.2)	1708	14.2 (12.4; 16.3)	2001	14.6 (12.6; 16.7)	1921	-1.1 (-1.7; -0.6)
Ecuador (2018)	Bottom 40%	19.8 (17.0; 23.0)	1476	22.6 (19.5; 26.1)	1843	28.4 (25.5; 31.5)	2377	31.9 (29.2; 34.8)	2726	33.2 (30.7; 35.8)	3087	17.4 (15.3; 19.8)	3644	-0.0 (-0.7; 0.6)
	Top 60%	12.2 (10.2; 14.7)	2059	16.2 (13.7; 19.2)	2564	16.6 (14.2; 19.4)	3236	16.2 (14.2; 18.6)	3563	15.5 (13.2; 18.0)	3630	6.4 (5.3; 7.8)	3418	-1.6 (-2.1; -1.2)
El Salvador (2014)	Bottom 40%	29.7 (24.9; 34.9)	402	27.8 (23.6; 32.4)	519	31.3 (26.9; 36.0)	640	33.1 (29.0; 37.6)	802	28.0 (24.4; 31.8)	891	26.1 (23.0; 29.3)	1123	-0.7 (-1.6; 0.3)
	Top 60%	21.3 (17.9; 25.2)	747	20.3 (17.0; 24.2)	791	19.9 (16.8; 23.5)	1036	20.0 (16.8; 23.5)	1116	13.6 (11.3; 16.4)	1304	13.4 (11.3; 15.8)	1499	-1.8 (-2.5; -1.1)
Guatemala (2014)	Bottom 40%	29.1 (25.2; 33.3)	733	28.9 (25.4; 32.7)	820	28.8 (26.2; 31.5)	1054	29.3 (26.5; 32.2)	1286	29.5 (26.7; 32.4)	1474	30.0 (27.5; 32.5)	1876	0.2 (-0.5; 1.0)
	Top 60%	21.5 (18.9; 24.3)	1413	20.4 (18.1; 22.8)	1696	21.4 (19.3; 23.6)	2023	21.6 (19.8; 23.5)	2295	16.1 (14.5; 17.9)	2523	14.2 (12.8; 15.9)	2950	-1.6 (-2.1; -1.2)
Guyana (2019)	Bottom 40%	28.2 (21.6; 36.0)	318	29.3 (21.0; 39.3)	345	28.8 (21.9; 36.7)	348	30.6 (24.7; 37.1)	368	21.4 (16.6; 27.3)	483	22.4 (17.3; 28.3)	559	-1.6 (-3.2; -0.1)
	Top 60%	20.6 (15.7; 26.4)	337	15.5 (11.4; 20.8)	343	18.2 (13.6; 23.9)	348	16.8 (12.1; 22.9)	357	11.1 (7.4; 16.3)	529	8.8 (5.9; 12.8)	544	-2.2 (-3.2; -1.2)
Haiti (2016)	Bottom 40%	19.1 (15.6; 23.1)	563	22.0 (18.0; 26.6)	556	20.0 (16.4; 24.2)	673	22.4 (18.9; 26.3)	677	18.9 (15.9; 22.4)	766	18.8 (16.1; 21.9)	1036	-0.3 (-1.1; 0.5)
	Top 60%	13.5 (10.4; 17.3)	654	14.6 (12.0; 17.7)	752	13.8 (11.5; 16.5)	972	13.8 (11.6; 16.3)	1242	12.0 (9.9; 14.5)	1436	11.2 (9.6; 13.1)	1737	-0.7 (-1.3; -0.1)
Honduras (2019)	Bottom 40%	23.7 (20.6; 27.1)	799	31.8 (28.3; 35.6)	960	32.6 (29.4; 35.9)	1044	34.6 (31.3; 38.0)	1165	32.8 (30.0; 35.8)	1325	37.8 (34.6; 41.1)	1431	2.1 (1.4; 2.9)
	Top 60%	20.2 (17.6; 23.2)	1055	22.5 (20.0; 25.3)	1295	24.5 (21.8; 27.4)	1342	20.9 (18.5; 23.6)	1460	19.2 (17.0; 21.7)	1716	19.7 (17.7; 22.0)	1934	-0.5 (-1.1; 0.1)
Jamaica (2011)	Bottom 40%	39.4 (31.5; 47.9)	214	35.7 (28.3; 43.9)	228	32.6 (26.4; 39.5)	234	41.9 (35.0; 49.1)	251	31.2 (25.4; 37.8)	285	21.5 (16.9; 27.0)	297	-3.2 (-4.8; -1.7)
	Top 60%	21.3 (16.9; 26.5)	381	20.7 (16.1; 26.2)	431	16.5 (13.3; 20.4)	472	13.4 (9.8; 17.9)	431	12.0 (8.6; 16.5)	437	10.2 (7.4; 14.0)	456	-2.3 (-3.3; -1.4)
Mexico (2015)	Bottom 40%	18.9 (14.0; 24.9)	496	24.5 (19.6; 30.0)	627	20.6 (16.8; 25.1)	720	27.4 (22.4; 33.1)	942	21.3 (17.1; 26.2)	1101	28.4 (24.1; 33.1)	1189	1.3 (0.1; 2.5)
	Top 60%	10.0 (6.9; 14.2)	618	12.7 (9.2; 17.4)	693	10.4 (7.9; 13.6)	841	12.8 (9.1; 17.8)	931	13.3 (9.4; 18.6)	1070	14.9 (11.4; 19.3)	1091	0.9 (-0.1; 1.8)
Paraguay (2016)	Bottom 40%	22.3 (15.9; 30.3)	255	29.8 (22.9; 37.9)	257	32.0 (26.1; 38.6)	339	31.8 (26.1; 38.0)	433	24.2 (19.8; 29.1)	521	25.3 (20.5; 30.7)	590	-0.5 (-1.9; 1.0)

Survey	Level	Women who had their first birth before 18 years by age groups												AAC (95%CI)
		45-49 years		40-44 years		35-39 years		30-34 years		25-29 years		20-24 years		
		% (95%CI)	N	% (95%CI)	N	% (95%CI)	N	% (95%CI)	N	% (95%CI)	N	% (95%CI)	N	
	Top 60%	9.8 (6.7; 14.1)	324	11.2 (7.9; 15.5)	397	13.3 (9.6; 18.1)	555	10.6 (7.7; 14.2)	817	10.9 (8.2; 14.3)	772	9.2 (7.0; 12.1)	752	-0.3 (-1.0; 0.5)
Peru (2020)	Bottom 40%	20.8 (18.3; 23.5)	1630	20.4 (17.9; 23.1)	2097	23.8 (21.3; 26.3)	2511	23.4 (21.3; 25.7)	2716	24.9 (22.6; 27.3)	2758	21.6 (19.4; 23.9)	2461	0.4 (-0.1; 1.0)
	Top 60%	9.3 (7.2; 11.9)	1430	9.9 (8.0; 12.3)	1885	9.1 (7.4; 11.1)	2358	8.0 (6.5; 9.8)	2437	9.2 (7.6; 11.1)	2419	5.9 (4.7; 7.3)	2204	-0.7 (-1.2; -0.3)
Saint Lucia (2012)	Bottom 40%	37.8 (24.8; 52.8)	59	31.4 (22.2; 42.3)	70	23.6 (13.8; 37.4)	46	36.3 (23.4; 51.4)	58	25.5 (15.0; 39.8)	77	22.6 (13.5; 35.2)	67	-2.2 (-5.1; 0.6)
	Top 60%	16.3 (10.2; 24.9)	113	14.4 (8.9; 22.4)	113	11.3 (6.0; 20.3)	112	8.7 (3.9; 18.3)	102	2.2 (0.6; 7.4)	101	2.8 (0.9; 8.0)	122	-3.0 (-4.2; -1.8)
Suriname (2018)	Bottom 40%	28.3 (22.5; 35.0)	306	28.8 (22.4; 36.0)	315	26.7 (19.3; 35.7)	397	28.1 (23.3; 33.5)	451	22.7 (18.3; 27.8)	529	24.8 (19.9; 30.5)	445	-1.0 (-2.4; 0.4)
	Top 60%	13.6 (10.1; 18.2)	426	7.4 (4.9; 11.0)	441	9.5 (6.6; 13.4)	573	9.7 (6.8; 13.5)	696	6.7 (4.5; 9.7)	609	6.2 (4.4; 8.7)	597	-1.0 (-1.6; -0.3)
Trinidad and Tobago (2011)	Bottom 40%	16.1 (11.3; 22.5)	214	13.8 (9.4; 19.9)	184	14.2 (9.8; 20.2)	218	14.3 (9.9; 20.1)	228	13.7 (9.6; 19.2)	293	13.0 (9.3; 18.0)	273	-0.4 (-1.6; 0.7)
	Top 60%	10.0 (7.2; 13.7)	392	8.2 (5.5; 12.0)	300	9.5 (6.6; 13.3)	331	4.1 (2.3; 7.2)	368	2.8 (1.6; 5.1)	404	1.9 (0.9; 4.0)	359	-1.7 (-2.3; -1.2)

AAC = average absolute change in percentage points; negative values indicate a reduction in the percentage of women who had a first birth before 18 years over generations whereas positive numbers indicate an increase in the figures.

Supplementary Table S5 – Early childbearing (percentage of women who had their first birth before 18 years) by generation, unweighted sample size, and corresponding average absolute change across groups by urban/rural residence, in Latin American and Caribbean countries.

Survey	Level	Women who had their first birth before 18 years by age groups												AAC (95%CI)
		45-49 years		40-44 years		35-39 years		30-34 years		25-29 years		20-24 years		
		% (95%CI)	N	% (95%CI)	N	% (95%CI)	N	% (95%CI)	N	% (95%CI)	N	% (95%CI)	N	
Argentina (2019)	Urban	NA	NA	NA	NA	NA	NA	NA	NA	NA	NA	NA	NA	NA
	Rural	NA	NA	NA	NA	NA	NA	NA	NA	NA	NA	NA	NA	NA
Barbados (2012)	Urban	15.3 (9.3; 24.0)	152	7.5 (4.2; 13.2)	128	12.7 (7.6; 20.5)	130	6.6 (3.5; 12.1)	149	11.3 (7.0; 17.6)	139	6.9 (3.4; 13.6)	109	-0.7 (-1.9; 0.6)
	Rural	12.9 (7.2; 22.1)	92	17.8 (9.0; 32.1)	80	13.3 (7.9; 21.7)	100	8.6 (3.7; 18.9)	95	4.5 (1.7; 11.4)	90	6.5 (2.7; 14.9)	83	-2.1 (-3.7; -0.5)
Belize (2015)	Urban	23.5 (16.5; 32.4)	181	20.6 (15.0; 27.6)	208	25.9 (19.5; 33.6)	248	21.2 (16.4; 27.0)	341	15.0 (11.1; 19.9)	396	16.6 (13.0; 21.0)	393	-1.7 (-3.0; -0.4)
	Rural	23.9 (17.2; 32.2)	208	27.3 (20.3; 35.5)	224	23.1 (16.9; 30.9)	302	20.8 (16.4; 26.2)	399	23.9 (18.8; 29.8)	421	17.9 (13.9; 22.7)	479	-1.4 (-2.8; 0.1)
Bolivia (2016)	Urban	17.8 (14.3; 22.0)	626	14.9 (12.0; 18.4)	829	12.7 (10.5; 15.4)	1020	15.2 (12.9; 17.8)	1100	13.8 (11.5; 16.4)	1224	13.2 (11.2; 15.6)	1323	-0.5 (-1.2; 0.2)
	Rural	20.0 (14.0; 27.8)	491	17.2 (13.6; 21.5)	516	23.7 (19.1; 29.1)	574	25.2 (21.3; 29.7)	586	22.9 (18.8; 27.5)	571	31.4 (25.9; 37.5)	543	2.3 (1.0; 3.6)
Colombia (2015)	Urban	12.1 (10.7; 13.7)	3243	14.3 (12.7; 16.2)	3124	17.0 (14.9; 19.2)	3478	19.7 (17.5; 21.9)	3819	16.9 (14.8; 19.1)	4012	15.9 (14.3; 17.6)	4464	0.8 (0.4; 1.2)
	Rural	22.9 (19.7; 26.4)	1072	26.1 (22.8; 29.7)	1149	29.9 (26.4; 33.7)	1177	31.2 (27.9; 34.7)	1257	32.1 (28.7; 35.6)	1248	32.0 (28.4; 35.7)	1332	1.9 (1.0; 2.7)
Costa Rica (2018)	Urban	13.3 (10.3; 17.0)	483	18.3 (13.8; 23.9)	529	20.6 (16.6; 25.1)	669	13.4 (10.0; 17.8)	811	11.6 (8.9; 15.0)	820	12.6 (10.0; 15.9)	772	-0.7 (-1.5; 0.1)
	Rural	14.9 (10.2; 21.3)	243	26.7 (21.0; 33.3)	293	19.7 (14.4; 26.5)	405	22.2 (16.5; 29.1)	483	20.6 (16.3; 25.7)	535	14.5 (11.1; 18.8)	463	-0.7 (-1.9; 0.4)
Cuba (2019)	Urban	12.1 (9.4; 15.4)	853	10.9 (7.6; 15.3)	662	9.7 (6.7; 13.9)	811	8.6 (6.3; 11.5)	1257	7.9 (5.6; 11.0)	1251	9.0 (6.4; 12.4)	920	-0.8 (-1.5; -0.0)
	Rural	25.8 (19.0; 34.0)	346	22.1 (14.3; 32.4)	313	14.1 (8.0; 23.5)	284	22.3 (15.7; 30.8)	435	8.0 (5.1; 12.1)	522	13.6 (9.3; 19.5)	389	-3.0 (-4.5; -1.5)
Dominican Republic (2019)	Urban	18.6 (16.3; 21.2)	1762	21.3 (19.0; 23.8)	1838	25.7 (23.4; 28.2)	2008	21.8 (19.4; 24.4)	2134	18.7 (16.7; 20.8)	2548	19.4 (17.4; 21.5)	2597	-0.3 (-0.9; 0.2)
	Rural	28.4 (23.9; 33.3)	758	27.6 (23.9; 31.5)	846	33.7 (28.6; 39.2)	899	35.9 (30.2; 41.9)	904	26.4 (22.4; 30.9)	1070	24.2 (20.5; 28.3)	1104	-0.8 (-1.8; 0.2)
Ecuador (2018)	Urban	14.1 (12.1; 16.3)	2258	18.2 (15.7; 21.0)	2792	18.6 (16.3; 21.2)	3641	19.4 (17.3; 21.5)	4065	19.6 (17.5; 22.0)	4376	9.0 (7.6; 10.5)	4357	-1.2 (-1.6; -0.7)
	Rural	16.2 (13.0; 20.0)	1277	19.0 (15.7; 22.9)	1615	27.3 (24.0; 30.8)	1972	28.4 (25.3; 31.8)	2224	28.3 (25.1; 31.8)	2341	15.6 (13.4; 18.1)	2705	-0.2 (-0.9; 0.6)
El Salvador (2014)	Urban	21.9 (18.5; 25.7)	744	20.2 (16.9; 24.0)	785	21.2 (17.9; 24.9)	1020	23.0 (19.5; 26.9)	1130	15.8 (13.2; 18.7)	1318	15.3 (13.1; 17.8)	1437	-1.4 (-2.1; -0.7)
	Rural	29.0 (24.3; 34.3)	405	28.4 (24.0; 33.2)	525	29.4 (25.1; 34.1)	656	29.3 (25.4; 33.5)	788	25.1 (21.9; 28.6)	877	22.7 (20.0; 25.6)	1185	-1.4 (-2.4; -0.5)
Guatemala (2014)	Urban	22.4 (19.5; 25.6)	1019	19.7 (17.1; 22.6)	1177	19.5 (17.0; 22.1)	1388	19.4 (17.3; 21.7)	1596	17.2 (15.0; 19.6)	1738	14.2 (12.3; 16.4)	2045	-1.5 (-2.0; -0.9)
	Rural	25.6 (22.4; 29.1)	1127	26.2 (23.5; 29.1)	1339	27.7 (25.5; 30.0)	1689	28.5 (26.4; 30.7)	1985	24.1 (22.0; 26.4)	2259	24.8 (22.8; 27.0)	2781	-0.5 (-1.1; 0.2)
Guyana (2019)	Urban	21.1 (14.3; 29.9)	179	15.0 (10.1; 21.8)	178	21.8 (15.5; 29.6)	228	17.0 (12.1; 23.3)	225	8.9 (5.5; 14.1)	296	11.9 (7.9; 17.4)	310	-2.0 (-3.4; -0.6)
	Rural	23.7 (19.1; 28.9)	476	22.0 (16.8; 28.2)	510	21.8 (16.9; 27.5)	468	23.5 (18.5; 29.3)	500	16.9 (13.2; 21.5)	716	14.0 (10.2; 19.1)	793	-1.9 (-3.0; -0.7)
Haiti (2016)	Urban	13.0 (9.7; 17.1)	411	15.1 (11.9; 19.0)	498	11.7 (9.1; 14.9)	638	12.8 (10.4; 15.7)	826	12.8 (10.0; 16.3)	948	9.4 (7.5; 11.7)	1181	-0.8 (-1.5; -0.2)
	Rural	17.4 (14.3; 21.0)	806	19.0 (16.0; 22.3)	810	19.3 (16.5; 22.5)	1007	19.6 (17.2; 22.3)	1093	15.0 (12.7; 17.6)	1254	17.6 (15.5; 20.0)	1592	-0.4 (-1.0; 0.3)
Honduras (2019)	Urban	18.9 (16.0; 22.3)	805	22.8 (19.9; 25.9)	928	22.7 (19.6; 26.1)	963	21.8 (19.0; 25.0)	1076	19.6 (16.8; 22.6)	1287	19.6 (17.3; 22.2)	1410	-0.3 (-1.0; 0.4)
	Rural	24.1 (21.3; 27.2)	1049	28.4 (25.4; 31.7)	1327	31.7 (29.0; 34.6)	1423	29.6 (26.9; 32.4)	1549	28.4 (26.0; 30.9)	1754	31.7 (29.2; 34.4)	1955	1.0 (0.3; 1.6)
Jamaica (2011)	Urban	23.0 (17.3; 29.9)	349	23.9 (18.3; 30.6)	413	19.3 (15.7; 23.6)	467	19.9 (15.2; 25.7)	459	17.4 (12.9; 23.1)	441	13.7 (10.4; 18.0)	476	-1.9 (-3.1; -0.8)
	Rural	33.1 (26.9; 40.0)	246	28.1 (22.6; 34.2)	246	26.3 (20.9; 32.5)	239	29.9 (23.4; 37.3)	223	22.3 (17.2; 28.5)	281	16.4 (12.6; 21.1)	277	-3.0 (-4.3; -1.7)
Mexico (2015)	Urban	11.1 (7.9; 15.5)	771	15.8 (12.3; 20.1)	894	12.1 (9.7; 14.9)	1059	15.6 (11.9; 20.2)	1251	15.8 (12.2; 20.2)	1426	18.9 (16.0; 22.2)	1552	1.3 (0.5; 2.2)
	Rural	19.4 (13.5; 27.1)	343	22.1 (16.4; 29.1)	426	21.9 (16.8; 28.0)	502	29.9 (23.3; 37.4)	622	19.3 (14.1; 25.8)	745	26.7 (20.9; 33.4)	728	0.9 (-0.6; 2.4)
Paraguay (2016)	Urban	12.5 (8.4; 18.2)	312	14.6 (10.3; 20.2)	367	18.7 (14.2; 24.2)	527	13.7 (10.3; 18.0)	781	13.5 (10.4; 17.3)	787	10.9 (8.6; 13.9)	779	-0.7 (-1.6; 0.2)
	Rural	16.5 (12.5; 21.5)	267	20.8 (15.9; 26.8)	287	21.3 (16.9; 26.4)	367	25.2 (20.2; 30.9)	469	19.4 (15.3; 24.3)	506	22.9 (18.5; 27.9)	563	0.9 (-0.2; 2.0)
Peru (2020)	Urban	11.3 (9.4; 13.6)	2006	11.9 (10.0; 14.1)	2626	12.4 (10.8; 14.2)	3440	10.9 (9.5; 12.5)	3646	12.5 (11.0; 14.1)	3659	8.8 (7.6; 10.2)	3294	-0.5 (-0.9; -0.1)
	Rural	21.8 (18.8; 25.1)	1054	21.3 (18.5; 24.4)	1356	25.7 (22.8; 28.8)	1429	28.1 (25.2; 31.1)	1507	26.0 (23.1; 29.1)	1518	24.4 (21.8; 27.4)	1371	0.8 (0.1; 1.5)
Saint Lucia (2012)	Urban	28.0 (18.9; 39.4)	57	25.7 (16.1; 38.5)	80	13.6 (6.5; 26.4)	63	21.7 (13.3; 33.2)	54	19.8 (11.8; 31.3)	63	18.4 (12.0; 27.0)	66	-1.5 (-3.6; 0.7)
	Rural	22.7 (15.4; 32.1)	115	19.0 (12.8; 27.4)	103	15.3 (8.9; 24.9)	95	17.4 (10.9; 26.7)	106	10.4 (5.2; 19.9)	115	7.4 (4.0; 13.3)	123	-3.0 (-4.5; -1.5)

Survey	Level	Women who had their first birth before 18 years by age groups												AAC (95%CI)
		45-49 years		40-44 years		35-39 years		30-34 years		25-29 years		20-24 years		
		% (95%CI)	N	% (95%CI)	N	% (95%CI)	N	% (95%CI)	N	% (95%CI)	N	% (95%CI)	N	
Suriname (2018)	Urban	15.5 (11.7; 20.2)	429	11.6 (8.3; 16.1)	451	11.6 (7.4; 17.8)	584	12.5 (9.5; 16.3)	736	10.3 (7.4; 14.2)	700	10.5 (8.0; 13.7)	623	-0.7 (-1.6; 0.1)
	Rural	28.7 (23.2; 35.0)	303	24.8 (18.9; 31.8)	305	28.4 (22.8; 34.6)	386	30.6 (25.4; 36.4)	411	25.0 (20.5; 30.2)	438	21.4 (17.3; 26.1)	419	-1.2 (-2.5; 0.0)
Trinidad and Tobago (2011)	Urban	11.5 (8.1; 16.1)	266	8.6 (5.6; 12.8)	225	10.7 (7.0; 16.1)	226	7.6 (5.0; 11.5)	270	7.3 (4.8; 11.0)	300	4.6 (2.7; 7.6)	279	-1.2 (-2.0; -0.5)
	Rural	12.5 (8.9; 17.1)	340	12.2 (8.6; 17.0)	259	11.7 (8.6; 15.7)	323	7.7 (4.8; 12.1)	326	6.9 (4.5; 10.5)	397	8.5 (5.5; 13.0)	353	-1.2 (-2.1; -0.3)

AAC = average absolute change in percentage points; negative values indicate a reduction in the percentage of women who had a first birth before 18 years over generations whereas positive numbers indicate an increase in the figures.

Supplementary Table S6 – Early childbearing (percentage of women who had their first birth before 18 years) by generation, unweighted sample sizes, and corresponding average absolute change across groups, by ethnicity, in Latin American and Caribbean countries.

Survey	Level	Women who had their first birth before 18 years by age groups												AAC (95%CI)
		45-49 years		40-44 years		35-39 years		30-34 years		25-29 years		20-24 years		
		% (95%CI)	N	% (95%CI)	N	% (95%CI)	N	% (95%CI)	N	% (95%CI)	N	% (95%CI)	N	
Argentina (2019)	Indigenous	29.3 (16.7; 46.3)	76	18.9 (9.0; 35.4)	86	18.0 (10.5; 29.2)	106	20.6 (11.8; 33.4)	110	11.5 (5.5; 22.3)	125	11.1 (4.9; 23.3)	109	-3.0 (-5.7; -0.4)
	Afrodescendants	NA	NA	NA	NA	NA	NA	NA	NA	NA	NA	NA	NA	NA
	Other	14.5 (11.6; 18.0)	1062	13.1 (10.6; 16.2)	1390	16.3 (13.4; 19.6)	1656	12.3 (10.1; 14.9)	1791	15.2 (12.7; 18.2)	1945	14.4 (12.1; 17.1)	1841	0.1 (-0.6; 0.7)
Barbados (2012)	Indigenous	NA	NA	NA	NA	NA	NA	NA	NA	NA	NA	NA	NA	NA
	Afrodescendants	NA	NA	NA	NA	NA	NA	NA	NA	NA	NA	NA	NA	NA
	Other	NA	NA	NA	NA	NA	NA	NA	NA	NA	NA	NA	NA	NA
Belize (2015)	Indigenous	43.4 (28.7; 59.4)	47	29.0 (15.7; 47.3)	55	34.7 (22.5; 49.3)	68	20.8 (12.2; 33.3)	90	29.5 (19.4; 42.1)	108	15.6 (9.9; 23.7)	132	-4.7 (-7.4; -2.0)
	Afrodescendants	22.1 (13.8; 33.6)	101	24.5 (16.3; 35.0)	124	20.6 (13.6; 30.0)	167	18.3 (13.0; 25.1)	234	17.0 (11.8; 23.9)	252	17.8 (12.6; 24.5)	249	-1.3 (-3.1; 0.6)
	Other	22.7 (16.4; 30.6)	203	25.0 (18.5; 32.9)	210	25.9 (19.5; 33.7)	275	23.3 (18.4; 29.1)	350	19.2 (14.9; 24.5)	391	18.3 (14.5; 22.9)	413	-1.4 (-2.8; -0.0)
Bolivia (2016)	Indigenous	16.3 (12.7; 20.5)	537	13.3 (10.3; 17.1)	555	16.5 (13.2; 20.5)	612	20.6 (17.0; 24.8)	569	22.4 (18.4; 27.0)	499	27.4 (22.7; 32.6)	416	2.4 (1.4; 3.4)
	Afrodescendants	NA	NA	NA	NA	NA	NA	NA	NA	NA	NA	NA	NA	NA
	Other	20.6 (15.7; 26.6)	577	16.9 (13.7; 20.7)	784	15.2 (12.6; 18.2)	975	16.7 (14.3; 19.5)	1112	13.7 (11.5; 16.2)	1281	14.9 (12.5; 17.6)	1443	-0.8 (-1.6; 0.0)
Colombia (2015)	Indigenous	17.9 (12.7; 24.7)	413	25.0 (19.3; 31.8)	444	24.3 (18.7; 30.9)	479	23.4 (18.1; 29.7)	543	27.4 (21.6; 33.9)	557	30.9 (25.0; 37.5)	671	2.0 (0.6; 3.5)
	Afrodescendants	19.6 (15.2; 24.9)	479	20.7 (15.7; 26.8)	485	25.6 (20.6; 31.4)	480	27.5 (22.5; 33.2)	613	23.9 (19.6; 28.8)	658	24.6 (19.5; 30.4)	625	1.1 (-0.2; 2.3)
	Other	13.3 (11.8; 15.0)	3420	15.6 (13.9; 17.4)	3344	18.6 (16.5; 20.8)	3693	21.2 (19.1; 23.3)	3917	19.1 (17.1; 21.3)	4041	18.2 (16.6; 19.9)	4499	1.1 (0.7; 1.5)
Costa Rica (2018)	Indigenous	29.3 (13.1; 53.3)	34	34.4 (17.0; 57.5)	33	26.1 (11.6; 48.8)	40	24.0 (8.2; 52.9)	36	15.1 (6.1; 32.6)	46	14.0 (6.7; 26.9)	59	-4.0 (-7.7; -0.3)
	Afrodescendants	22.5 (14.1; 33.8)	109	23.4 (15.6; 33.5)	141	18.2 (12.2; 26.3)	157	25.0 (16.6; 35.9)	241	14.5 (9.5; 21.5)	226	16.9 (11.8; 23.5)	226	-1.5 (-3.2; 0.3)
	Other	11.5 (8.6; 15.3)	533	20.1 (15.8; 25.2)	585	18.9 (15.2; 23.4)	785	12.7 (9.9; 16.2)	930	13.8 (10.8; 17.6)	975	12.2 (9.5; 15.5)	854	-0.4 (-1.2; 0.4)
Cuba (2019)	Indigenous	NA	NA	NA	NA	NA	NA	NA	NA	NA	NA	NA	NA	NA
	Afrodescendants	20.5 (15.1; 27.2)	408	17.0 (11.2; 25.0)	347	10.0 (5.7; 17.0)	364	12.1 (8.2; 17.4)	527	11.4 (7.4; 17.1)	542	12.2 (8.4; 17.4)	431	-1.4 (-2.6; -0.1)
	Other	15.2 (11.9; 19.2)	791	14.5 (9.5; 21.6)	628	12.0 (8.1; 17.4)	731	13.7 (10.4; 18.0)	1165	6.2 (4.3; 8.8)	1231	9.4 (6.8; 12.9)	878	-1.7 (-2.5; -0.8)
Dominican Republic (2019)	Indigenous	NA	NA	NA	NA	NA	NA	NA	NA	NA	NA	NA	NA	NA
	Afrodescendants	NA	NA	NA	NA	NA	NA	NA	NA	NA	NA	NA	NA	NA
	Other	NA	NA	NA	NA	NA	NA	NA	NA	NA	NA	NA	NA	NA
Ecuador (2018)	Indigenous	8.5 (5.5; 12.9)	423	17.6 (10.5; 27.9)	534	20.1 (14.8; 26.7)	728	24.8 (18.6; 32.3)	742	22.9 (17.6; 29.2)	836	9.3 (6.2; 13.7)	925	0.5 (-0.5; 1.5)
	Afrodescendants	23.3 (15.3; 33.7)	135	31.7 (22.4; 42.7)	154	34.0 (25.7; 43.5)	251	21.2 (14.2; 30.5)	238	25.5 (17.3; 36.0)	283	15.1 (10.3; 21.7)	300	-2.6 (-4.5; -0.8)
	Other	14.2 (12.3; 16.3)	2824	17.7 (15.4; 20.2)	3551	19.9 (17.9; 22.0)	4439	21.0 (19.1; 23.0)	5081	21.6 (19.6; 23.8)	5344	10.0 (8.7; 11.4)	5558	-0.9 (-1.4; -0.5)
El Salvador (2014)	Indigenous	NA	NA	NA	NA	NA	NA	NA	NA	NA	NA	NA	NA	NA
	Afrodescendants	NA	NA	NA	NA	NA	NA	NA	NA	NA	NA	NA	NA	NA
	Other	NA	NA	NA	NA	NA	NA	NA	NA	NA	NA	NA	NA	NA
Guatemala (2014)	Indigenous	25.8 (22.3; 29.7)	813	24.8 (21.5; 28.4)	902	25.5 (22.9; 28.4)	1172	25.0 (22.5; 27.7)	1331	22.1 (19.5; 24.9)	1502	21.6 (19.3; 24.0)	1850	-0.9 (-1.7; -0.2)
	Afrodescendants	NA	NA	NA	NA	NA	NA	NA	NA	NA	NA	NA	NA	NA
	Other	22.9 (20.2; 25.7)	1331	22.0 (19.7; 24.4)	1610	22.6 (20.5; 24.9)	1900	23.8 (21.9; 25.9)	2245	20.2 (18.3; 22.3)	2486	19.2 (17.4; 21.2)	2957	-0.7 (-1.3; -0.2)
Guyana (2019)	Indigenous	28.8 (19.0; 41.0)	102	22.3 (13.6; 34.3)	117	34.6 (25.5; 44.9)	169	37.3 (27.6; 48.2)	145	19.6 (13.4; 27.7)	196	34.1 (24.7; 44.9)	214	-0.2 (-2.5; 2.2)
	Afrodescendants	17.6 (12.2; 24.7)	166	15.6 (8.2; 27.6)	173	16.8 (10.5; 25.8)	183	17.1 (11.2; 25.2)	178	7.4 (4.6; 11.6)	264	8.0 (5.2; 12.1)	282	-2.3 (-3.5; -1.0)
	Other	24.9 (19.9; 30.8)	386	22.7 (17.5; 28.9)	395	22.5 (17.1; 28.9)	341	21.7 (16.2; 28.4)	399	17.7 (13.5; 22.8)	548	13.9 (9.5; 19.9)	604	-2.1 (-3.3; -0.8)
Haiti (2016)	Indigenous	NA	NA	NA	NA	NA	NA	NA	NA	NA	NA	NA	NA	NA
	Afrodescendants	NA	NA	NA	NA	NA	NA	NA	NA	NA	NA	NA	NA	NA

Survey	Level	Women who had their first birth before 18 years by age groups												AAC (95%CI)
		45-49 years		40-44 years		35-39 years		30-34 years		25-29 years		20-24 years		
		% (95%CI)	N	% (95%CI)	N	% (95%CI)	N	% (95%CI)	N	% (95%CI)	N	% (95%CI)	N	
	Other	NA	NA	NA	NA	NA	NA	NA	NA	NA	NA	NA	NA	NA
Honduras (2019)	Indigenous	20.5 (14.8; 27.5)	234	22.6 (17.4; 28.7)	284	19.1 (14.5; 24.7)	304	16.9 (12.5; 22.4)	344	23.9 (19.1; 29.5)	425	25.5 (20.3; 31.4)	437	0.8 (-0.6; 2.2)
	Afrodescendants	17.3 (5.0; 45.5)	39	19.2 (8.7; 37.3)	39	24.5 (10.8; 46.4)	44	21.8 (9.0; 44.2)	43	12.2 (4.5; 29.3)	51	14.6 (5.4; 33.8)	46	-1.4 (-5.1; 2.2)
	Other	22.0 (19.7; 24.5)	1514	26.2 (23.9; 28.7)	1856	28.4 (26.1; 30.9)	1952	27.3 (25.1; 29.6)	2144	24.5 (22.5; 26.6)	2451	26.3 (24.4; 28.3)	2771	0.4 (-0.2; 0.9)
Jamaica (2011)	Indigenous	NA	NA	NA	NA	NA	NA	NA	NA	NA	NA	NA	NA	NA
	Afrodescendants	NA	NA	NA	NA	NA	NA	NA	NA	NA	NA	NA	NA	NA
	Other	NA	NA	NA	NA	NA	NA	NA	NA	NA	NA	NA	NA	NA
Mexico (2015)	Indigenous	18.9 (11.7; 29.2)	126	30.7 (18.6; 46.2)	119	18.8 (12.0; 28.1)	150	29.0 (16.2; 46.3)	173	20.3 (12.8; 30.7)	190	21.8 (14.0; 32.3)	231	0.2 (-2.0; 2.4)
	Afrodescendants	NA	NA	NA	NA	NA	NA	NA	NA	NA	NA	NA	NA	NA
	Other	12.4 (9.2; 16.4)	988	16.3 (13.3; 19.8)	1201	13.5 (11.2; 16.3)	1409	17.9 (14.4; 22.1)	1697	16.4 (13.2; 20.2)	1979	20.4 (17.5; 23.6)	2049	1.3 (0.6; 2.1)
Paraguay (2016)	Indigenous	NA	NA	NA	NA	NA	NA	NA	NA	NA	NA	NA	NA	NA
	Afrodescendants	NA	NA	NA	NA	NA	NA	NA	NA	NA	NA	NA	NA	NA
	Other	NA	NA	NA	NA	NA	NA	NA	NA	NA	NA	NA	NA	NA
Peru (2020)	Indigenous	26.8 (17.9; 38.1)	192	19.6 (13.5; 27.6)	209	24.1 (17.5; 32.3)	216	28.2 (21.5; 36.1)	206	25.0 (17.7; 34.1)	205	25.6 (19.0; 33.6)	214	0.7 (-1.2; 2.7)
	Afrodescendants	NA	NA	NA	NA	NA	NA	NA	NA	NA	NA	NA	NA	NA
	Other	15.2 (12.0; 18.9)	1152	12.5 (10.3; 15.1)	1689	13.7 (11.8; 15.9)	2299	13.6 (11.8; 15.7)	2598	13.9 (12.1; 15.9)	2718	12.9 (11.1; 15.0)	2366	-0.1 (-0.7; 0.5)
Saint Lucia (2012)	Indigenous	NA	NA	NA	NA	NA	NA	NA	NA	NA	NA	NA	NA	NA
	Afrodescendants	NA	NA	NA	NA	NA	NA	NA	NA	NA	NA	NA	NA	NA
	Other	NA	NA	NA	NA	NA	NA	NA	NA	NA	NA	NA	NA	NA
Suriname (2018)	Indigenous	16.7 (5.8; 39.8)	35	37.5 (21.0; 57.6)	46	29.1 (16.2; 46.4)	55	28.0 (15.4; 45.4)	46	32.1 (19.7; 47.7)	65	26.6 (14.6; 43.4)	64	1.1 (-2.7; 4.8)
	Afrodescendants	30.1 (23.6; 37.5)	258	27.4 (20.5; 35.5)	280	25.7 (18.4; 34.6)	396	21.5 (17.2; 26.6)	447	18.6 (14.0; 24.4)	431	18.5 (14.7; 23.0)	463	-2.4 (-3.8; -1.0)
	Other	13.8 (10.3; 18.1)	425	7.1 (4.7; 10.6)	408	7.0 (4.7; 10.4)	490	12.0 (8.6; 16.4)	627	9.0 (6.4; 12.6)	617	8.1 (5.7; 11.3)	491	-0.3 (-1.0; 0.5)
Trinidad and Tobago (2011)	Indigenous	NA	NA	NA	NA	NA	NA	NA	NA	NA	NA	NA	NA	NA
	Afrodescendants	NA	NA	NA	NA	NA	NA	NA	NA	NA	NA	NA	NA	NA
	Other	NA	NA	NA	NA	NA	NA	NA	NA	NA	NA	NA	NA	NA

AAC = average absolute change in percentage points; negative values indicate a reduction in the percentage of women who had a first birth before 18 years over generations whereas positive numbers indicate an increase in the figures.

Supplementary Table S7 – Adolescent fertility rate (births per 1,000 women aged 15-19 years) by wealth (bottom 40% and top 60%) and p value for interaction between wealth and survey year based on country-specific variance weighted linear regression models.

Country	Year	Adolescent Fertility Rate (95%CI)		Interaction p value
		Bottom 40%	Top 60%	
Bolivia	1994	137.7 (119.4; 156.1)	78.4 (69.9; 86.8)	0.98
	1998	150.7 (135.0; 166.3)	64.7 (56.1; 73.3)	
	2003	162.5 (148.6; 176.5)	72.5 (65.6; 79.4)	
	2008	145.7 (132.4; 159.0)	64.0 (57.7; 70.4)	
	2016	118.1 (101.8; 134.4)	51.0 (44.8; 57.3)	
Colombia	1995	149.4 (134.9; 163.8)	62.3 (55.3; 69.3)	0.03
	2000	144.0 (131.0; 157.0)	56.4 (50.0; 62.8)	
	2005	140.2 (132.1; 148.4)	64.7 (58.9; 70.5)	
	2010	129.9 (123.0; 136.8)	58.0 (54.0; 61.9)	
	2015	120.8 (113.3; 128.4)	46.0 (40.9; 51.0)	
Dominican Republic	1996	192.2 (174.0; 210.4)	76.8 (66.5; 87.0)	<0.001
	1999	162.0 (121.1; 202.9)	67.7 (41.0; 94.5)	
	2002	183.5 (173.2; 193.8)	81.3 (73.3; 89.2)	
	2007	156.8 (147.4; 166.2)	65.3 (59.1; 71.5)	
	2013	138.5 (124.9; 152.0)	59.0 (49.5; 68.6)	
	2014	145.0 (136.3; 153.8)	60.0 (54.8; 65.2)	
	2019	131.8 (121.0; 142.7)	46.7 (40.6; 52.8)	
Ecuador	2012	145.7 (127.5; 163.8)	91.0 (80.3; 101.6)	0.61
	2018	101.4 (95.0; 107.8)	52.7 (47.1; 58.2)	
Guatemala	1995	188.3 (171.5; 205.2)	96.6 (84.4; 108.8)	<0.001
	1998	181.1 (164.1; 198.2)	92.8 (77.2; 108.5)	
	2014	130.9 (122.2; 139.6)	70.4 (64.9; 75.9)	
Guyana	2009	159.8 (121.9; 197.7)	58.1 (45.5; 70.7)	0.13
	2014	139.5 (119.1; 160.0)	37.7 (30.0; 45.5)	
	2019	116.3 (98.1; 134.5)	43.6 (32.5; 54.6)	
Haiti	1994	110.6 (89.9; 131.4)	65.8 (53.8; 77.8)	0.58
	2000	96.2 (76.3; 116.1)	73.0 (61.6; 84.4)	
	2005	91.6 (79.3; 104.0)	59.4 (51.7; 67.1)	
	2012	88.5 (78.2; 98.8)	55.3 (48.2; 62.3)	
	2016	86.7 (76.8; 96.7)	45.5 (39.5; 51.5)	
Honduras	2005	150.5 (142.0; 159.0)	88.2 (81.7; 94.7)	0.86
	2011	144.6 (136.7; 152.5)	75.4 (69.6; 81.2)	
	2019	137.1 (127.8; 146.3)	75.1 (68.9; 81.3)	
Peru	1996	144.4 (128.6; 160.1)	46.2 (41.0; 51.5)	<0.001
	2000	132.3 (120.7; 143.9)	42.0 (36.8; 47.3)	
	2004	108.8 (87.1; 130.4)	41.1 (31.8; 50.3)	
	2005	117.6 (81.3; 153.8)	39.9 (29.1; 50.7)	
	2006	124.9 (92.6; 157.2)	38.1 (27.9; 48.3)	
	2007	129.8 (103.8; 155.8)	44.7 (32.8; 56.5)	
	2008	127.8 (102.2; 153.3)	47.3 (40.5; 54.1)	
	2009	120.3 (106.9; 133.7)	43.1 (36.5; 49.6)	
	2010	114.6 (100.6; 128.6)	49.0 (41.3; 56.6)	
	2011	105.3 (92.4; 118.2)	39.5 (33.4; 45.7)	
	2012	114.2 (100.5; 127.9)	41.8 (35.4; 48.2)	
	2013	105.6 (92.3; 118.8)	46.1 (38.9; 53.2)	

Country	Year	Adolescent Fertility Rate (95%CI)		Interaction p value
		Bottom 40%	Top 60%	
	2014	104.3 (91.3; 117.4)	45.6 (39.5; 51.6)	
	2015	108.1 (98.7; 117.5)	39.4 (35.4; 43.4)	
	2016	107.9 (98.8; 117.1)	37.8 (33.7; 41.9)	
	2017	103.2 (93.3; 113.1)	39.4 (34.3; 44.5)	
	2018	94.2 (85.4; 102.9)	28.9 (25.2; 32.6)	
	2019	96.2 (86.9; 105.5)	29.3 (25.6; 33.1)	

Supplementary Table S8 – Annual average absolute change (AAC) in adolescent fertility rate (births per 1,000 women aged 15-19 years in the five years preceding the survey) over time at the national level and by wealth groups (bottom 40% and top 60%) and predicted values for 2030.

Country	Number of surveys	Level	Annual AAC (95%CI)	p value	2030 forecast estimate
Bolivia	5	National	-0.9 (-1.3; -0.4)	<0.001	61.6
Colombia	5	National	-0.7 (-1.0; -0.3)	<0.001	67.3
Dominican Republic	7	National	-1.7 (-2.1; -1.3)	<0.001	62.6
Ecuador	2	National	-6.5 (-8.7; -4.3)	<0.001	..
Guatemala	3	National	-1.8 (-2.4; -1.2)	<0.001	64.9
Guyana	3	National	-2.3 (-4.3; -0.4)	<0.001	44.6
Haiti	5	National	-0.9 (-1.4; -0.5)	<0.001	46.2
Honduras	3	National	-0.7 (-1.3; -0.2)	<0.001	89.4
Peru	18	National	-0.8 (-1.0; -0.6)	<0.001	45.5
Bolivia	5	Bottom 40%	-1.1 (-2.1; -0.2)	<0.001	102.4
		Top 60%	-1.1 (-1.5; -0.7)	<0.001	35.6
Colombia	5	Bottom 40%	-1.5 (-2.2; -0.9)	<0.001	97.8
		Top 60%	-0.7 (-1.0; -0.3)	<0.001	35.9
Dominican Republic	7	Bottom 40%	-2.8 (-3.4; -2.1)	<0.001	101.4
		Top 60%	-1.4 (-1.9; -1.0)	<0.001	30.8
Ecuador	2	Bottom 40%	-7.4 (-10.6; -4.2)	<0.001	12.9
		Top 60%	-6.4 (-8.4; -4.4)	<0.001	..
Guatemala	3	Bottom 40%	-3.1 (-3.9; -2.2)	<0.001	81.8
		Top 60%	-1.4 (-2.0; -0.8)	<0.001	48.2
Guyana	3	Bottom 40%	-4.4 (-8.2; -0.7)	<0.001	67.3
		Top 60%	-1.3 (-2.9; 0.4)	0.10	29.6
Haiti	5	Bottom 40%	-0.8 (-1.7; -0.0)	<0.001	74.9
		Top 60%	-1.1 (-1.6; -0.6)	<0.001	29.8
Honduras	3	Bottom 40%	-1.0 (-1.9; -0.1)	<0.001	126.6
		Top 60%	-0.9 (-1.5; -0.2)	<0.001	65.7
Peru	18	Bottom 40%	-1.9 (-2.4; -1.4)	<0.001	75.2
		Top 60%	-0.6 (-0.8; -0.4)	<0.001	22.4

Note: .. represents estimated values lower than zero; * annual AAC = average absolute change in number of births per 1,000 women-years; negative values indicate an annual reduction in the average number of births whereas positive numbers indicate an increase in the figures.

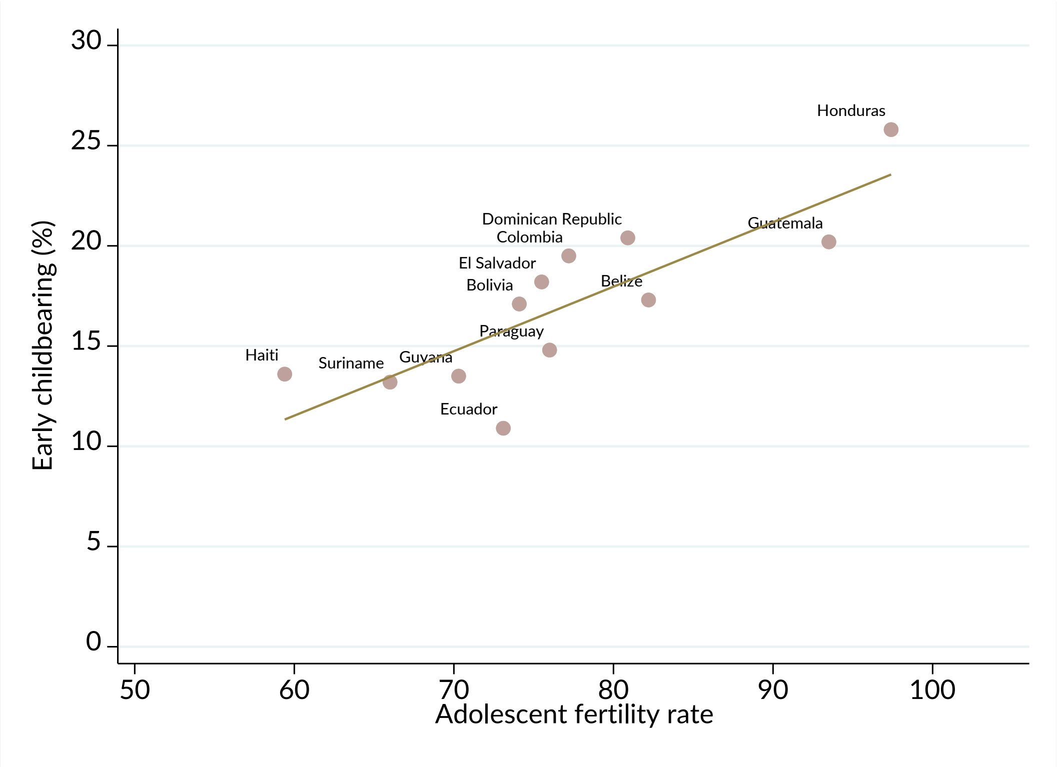

Supplementary Figure S1 – Early childbearing (percentage of women aged 20-24 years who had their first birth before 18 years) and adolescent fertility rates (births per 1,000 women aged 15-19 years in the five years preceding the survey) with a regression line (Pearson's correlation = 0·83, p = 0.001).

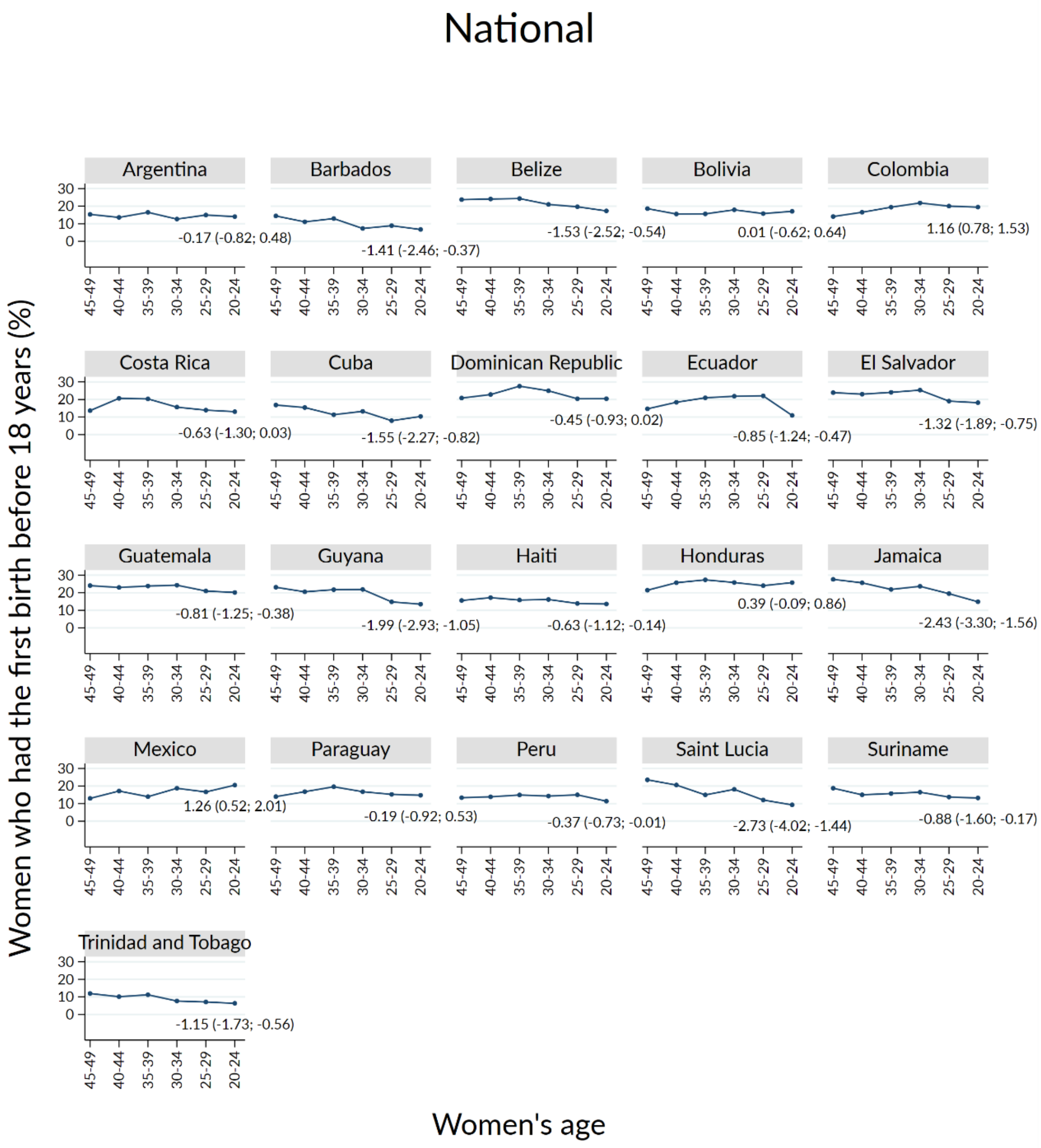

Supplementary Figure S2 – Average absolute change in early childbearing (women having a first birth before the age of 18) in percentage points across generations at the national level in Latin American and Caribbean countries.

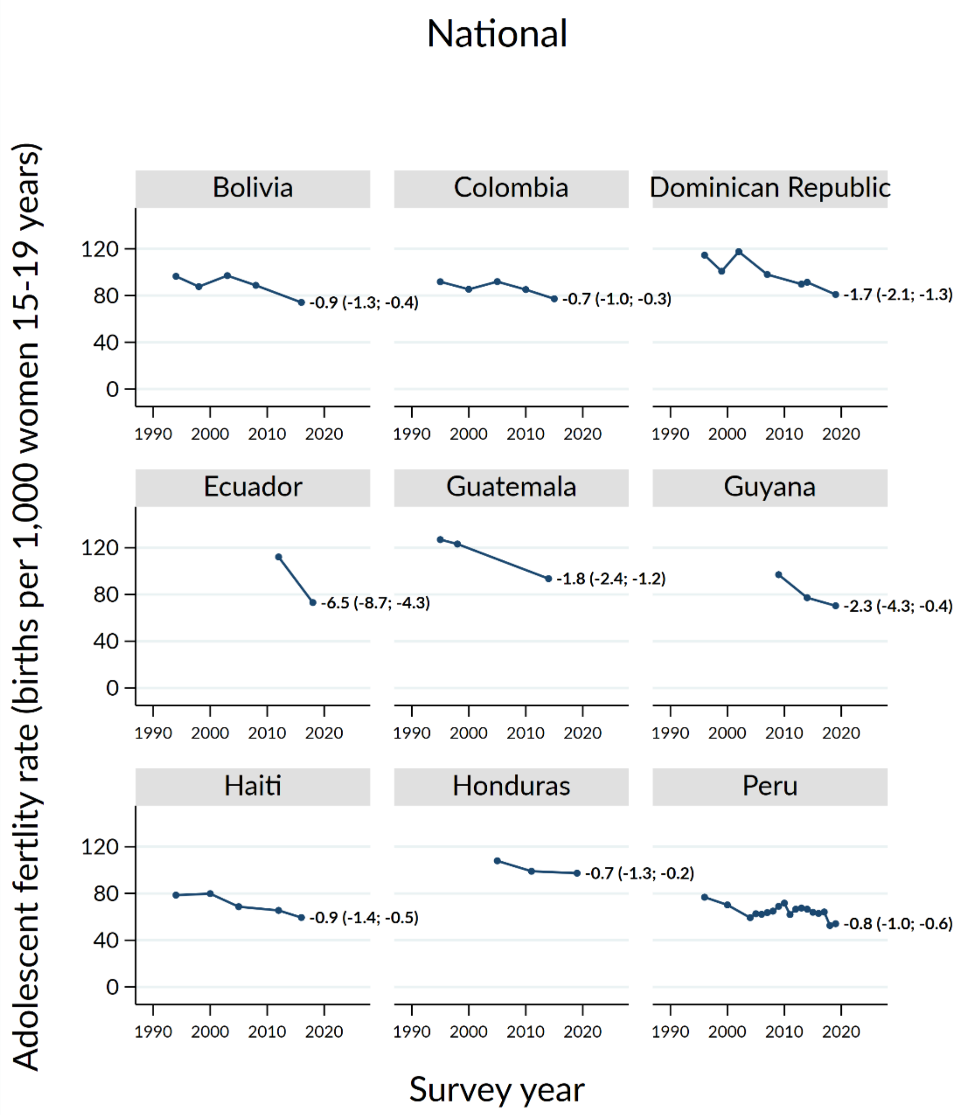

Supplementary Figure S3 – Trends in adolescent fertility rate (births per 1,000 women aged 15-19 years in the five years preceding the survey) at the national level and corresponding annual average absolute change in Latin American and Caribbean countries.

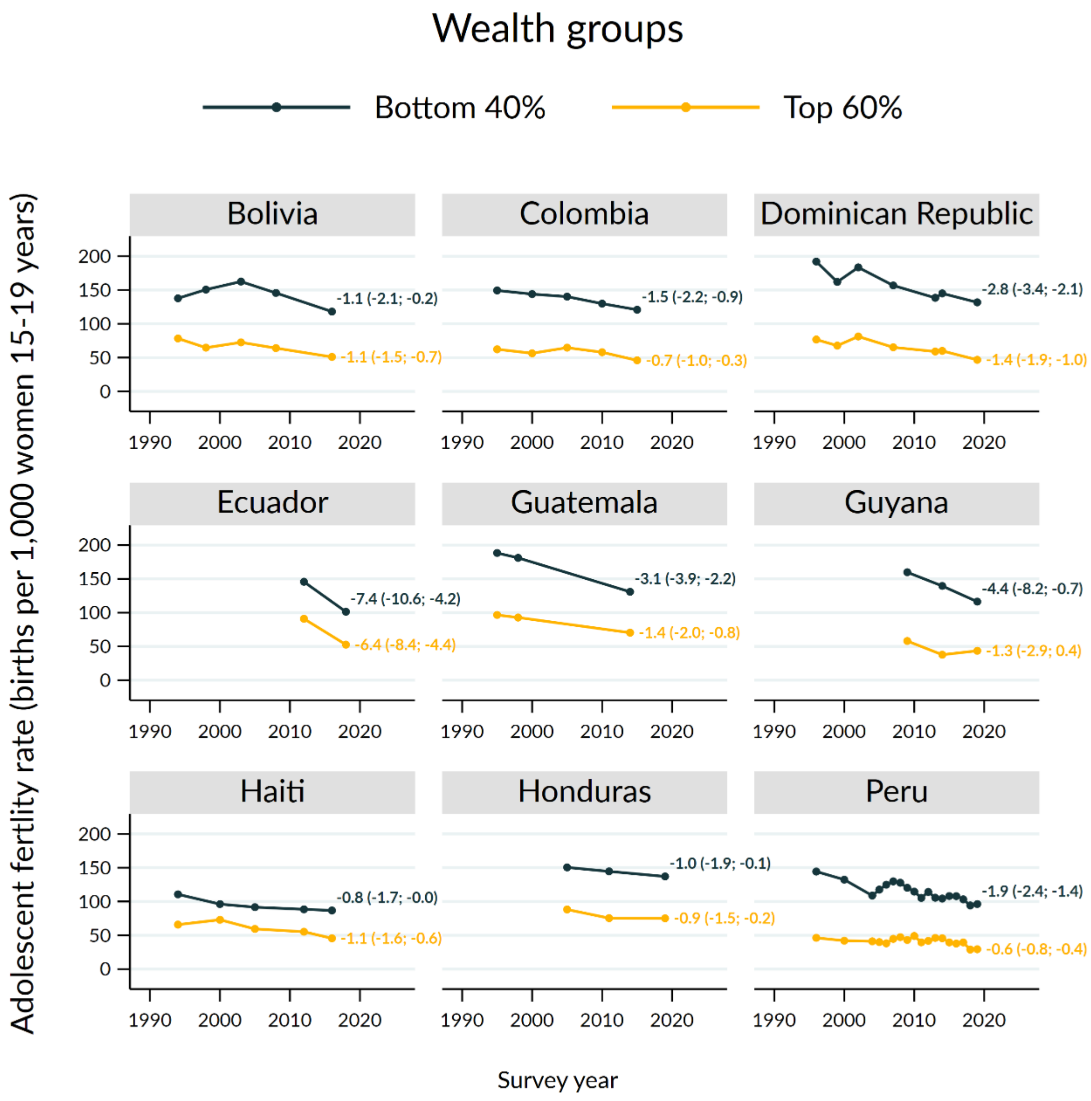

Supplementary Figure S4 – Trends in adolescent fertility rate (births per 1,000 women aged 15-19 years in the five years preceding the survey) by wealth groups and corresponding annual average absolute change in Latin American and Caribbean countries.

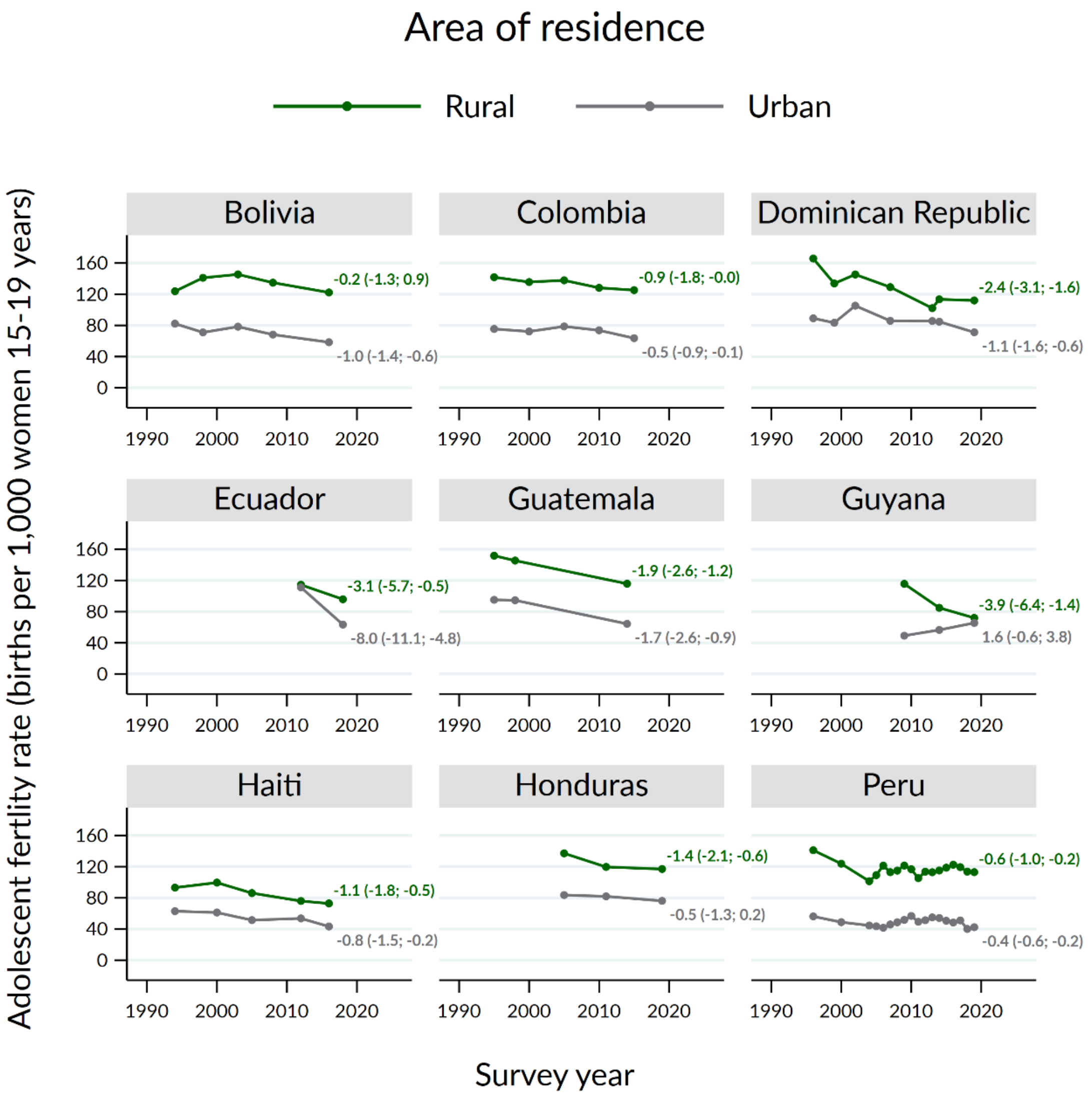

Supplementary Figure S5 – Trends in adolescent fertility rate (births per 1,000 women aged 15-19 years in the five years preceding the survey) by urban/rural residence and corresponding annual average absolute change in Latin American and Caribbean countries.

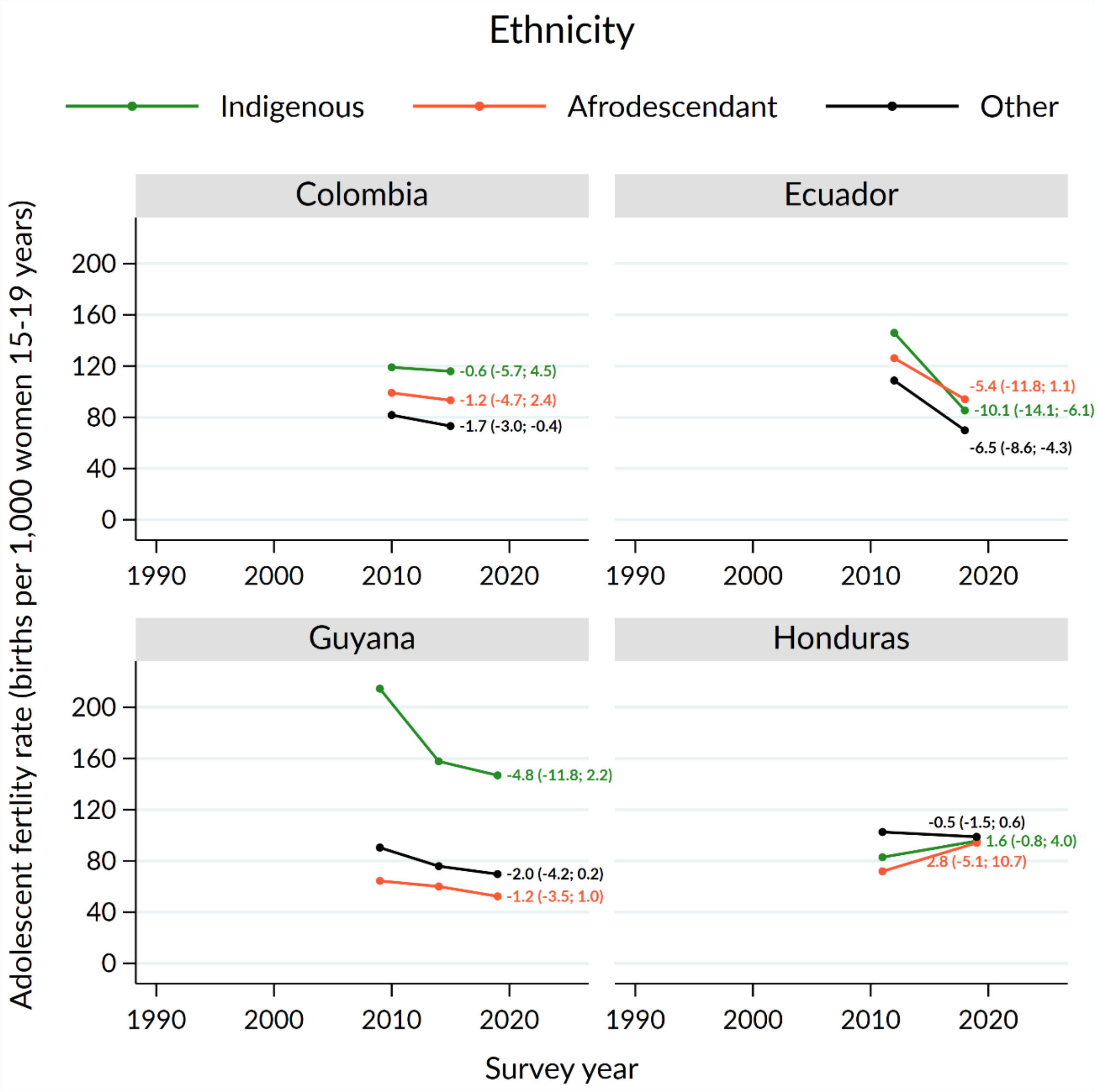

Supplementary Figure S6 – Trends in adolescent fertility rate (births per 1,000 women aged 15-19 years in the five years preceding the survey) by ethnicity (Indigenous, Afrodescendant, Other non-Indigenous/non-Afrodescendant groups) in Latin American and Caribbean countries.
